# Development of a physiologically relevant and easily scalable LUHMES cell-based model of G2019S LRRK2-driven Parkinson's disease

**DOI:** 10.1242/dmm.048017

**Published:** 2021-06-11

**Authors:** Barbara Calamini, Nathalie Geyer, Nathalie Huss-Braun, Annie Bernhardt, Véronique Harsany, Pierrick Rival, May Cindhuchao, Dietmar Hoffmann, Sabine Gratzer

**Affiliations:** 1 Molecular Discovery, Immuno-Oncology Therapeutic Research Area, Sanofi Strasbourg R&D Center, 16 rue d'Ankara, 67000 Strasbourg, France; 2 BioTherapeutics/e-Biology - Bioinformatics, Sanofi Biologics Research, 13 quai Jules Guesde, 94400 Vitry-sur-Seine, France; 3 Molecular Screening Technology, Sanofi Biologics Research, 270 Albany Street, Cambridge, MA 02139, USA

**Keywords:** Parkinson's disease, LUHMES cell, Leucine-rich repeat kinase 2, LRRK2, Translational disease model

## Abstract

Parkinson's disease (PD) is a fatal neurodegenerative disorder that is primarily caused by the degeneration and loss of dopaminergic neurons of the substantia nigra in the ventral midbrain. Mutations in leucine-rich repeat kinase 2 (*LRRK2*) are the most common genetic cause of late-onset PD identified to date, with G2019S being the most frequent LRRK2 mutation, which is responsible for up to 1-2% of sporadic PD and up to 6% of familial PD cases. As no treatment is available for this devastating disease, developing new therapeutic strategies is of foremost importance. Cellular models are commonly used for testing novel potential neuroprotective compounds. However, current cellular PD models either lack physiological relevance to dopaminergic neurons or are too complex and costly for scaling up the production process and for screening purposes. In order to combine biological relevance and throughput, we have developed a PD model in Lund human mesencephalic (LUHMES) cell-derived dopaminergic neurons by overexpressing wild-type (WT) and G2019S LRRK2 proteins. We show that these cells can differentiate into dopaminergic-like neurons and that expression of mutant LRRK2 causes a range of different phenotypes, including reduced nuclear eccentricity, altered mitochondrial and lysosomal morphologies, and increased dopaminergic cell death. This model could be used to elucidate G2019S LRRK2-mediated dopaminergic neural dysfunction and to identify novel molecular targets for disease intervention. In addition, our model could be applied to high-throughput and phenotypic screenings for the identification of novel PD therapeutics.

## Abstract (precis)

**Summary:** Using Lund human mesencephalic cell-derived dopaminergic neurons, we developed a translational model of Parkinson's disease (PD) for the study of PD biology and for high-throughput screening for the identification of small-molecule PD therapeutics.

## INTRODUCTION

Parkinson's disease (PD) is a progressive neurodegenerative disease for which no preventative or curative treatments exist. The histopathological hallmark of PD is the degeneration and loss of dopaminergic neurons of the substantia nigra in the ventral midbrain (Fearnley and Lees, 1991; Lees et al., 2009). Although most PD cases are sporadic, resulting from a complex interaction of environmental and genetic factors, rare familial forms of the disease also exist and are caused by mutations in genes such as leucine-rich repeat kinase 2 (*LRRK2*), α-synuclein (*SNCA*), *PARK2* (also known as *PRKN*), *PARK7* or PTEN-induced kinase 1 (*PINK1*) (Kalia and Lang, 2015; Martin et al., 2011). Of interest, genome-wide association studies (GWAS) have linked variations in some of these genes (e.g. *LRRK2* and *SNCA*) as risk factors for the development of non-familial PD (Cookson, 2015; Lill et al., 2012; Ross et al., 2011; Satake et al., 2009; Simon-Sanchez et al., 2009), suggesting that shared biological pathways drive disease pathogenesis in both hereditary and sporadic cases (Cookson, 2015; Kluss et al., 2019).

*LRRK2* mutations are the most common genetic cause of both familial and sporadic PD (Paisan-Ruiz et al., 2004; Zimprich et al., 2004). The *LRRK2* gene encodes a large, multidomain protein with kinase activity. Among the 20 identified LRRK2 mutations, six of them have been demonstrated to be pathogenic and causing toxicity in cellular and animal models (Rudenko and Cookson, 2014). The Gly2019Ser (G2019S) substitution in the LRRK2 kinase domain is the most common mutation, accounting for 5-6% of familial PD and 1-2% of sporadic cases (Correia Guedes et al., 2010). The toxic effects of LRRK2 G2019S on dopaminergic (DA) neurons are believed to result from increased kinase activity compared to the WT protein (West et al., 2005). LRRK2 is considered a very attractive target for therapeutic development, and, in fact, several potent and selective LRRK2 kinase inhibitors have been developed and are considered one of the major disease-modifying therapeutic strategies for PD (Taymans and Greggio, 2016).

In order to identify novel therapeutics and to understand the physiological function of LRRK2 and the molecular mechanism underlying the pathogenic role of LRRK2 mutations, relevant cellular models of LRRK2-mediated PD are needed. The unavailability of bona fide cellular models of LRRK2 driven-PD, in particular of living midbrain DA neurons from LRRK2 PD patients, poses big hurdles to the understanding of the LRRK2-mediated PD pathological mechanisms. Although the use of induced pluripotent stem cells (iPSCs), together with genome-editing technologies such as CRISPR/Cas9, has dramatically changed this aspect of *in vitro* PD modeling by offering easy access to non-immortalized human midbrain DA neurons and to patient-specific material, this system also presents several limitations (Beevers et al., 2013; Volpato and Webber, 2020; Weykopf et al., 2019). For example, iPSC-based studies are typically very costly and laborious, thus limiting the experimental sample sizes and the number of replicates achievable in a single study. iPSCs display high degrees of interindividual genetic variation (Volpato and Webber, 2020), thus requiring the use of various clones from one donor or the implementation of isogenic pairs. An additional challenge in developing iPSC-derived disease models is that the quality, quantity and purity of the desired cell population may vary depending on the *in vitro* differentiation protocols used (Weykopf et al., 2019). This may limit the number of cells available for large-scale studies and high-throughput screening (HTS) campaigns. Biologically relevant cellular models that can recapitulate multiple aspects of the desired *in vivo* system, but are also easy to culture and maintain, can be expanded to large scale and provide a good batch-to-batch consistency, are needed for deciphering the LRRK2 signaling pathways and to ensure efficient and meaningful HTS campaigns.

With this aim, we have developed a PD model by overexpressing wild-type (WT) and G2019S LRRK2 in a Lund human mesencephalic (LUHMES) cell line. LUHMES cells are derived from embryonic human mesencephalon and immortalized by the expression of the *v-myc* gene under the control of a tetracycline (TET)-inducible transcriptional activator (TET-off system) (Lotharius et al., 2005). Proliferating LUHMES cells can be differentiated into post-mitotic DA-like neurons by the addition of TET or its derivatives (e.g. doxycycline), cyclic AMP (cAMP) and glial-derived neurotrophic factor (GDNF) (Scholz et al., 2011). LUHMES cells offer a good alternative to iPSC-derived midbrain DA neurons as they possess physiological relevance, are easy to culture and are relatively inexpensive to maintain. In addition, they can be easily expanded to obtain large-scale culture and display good batch-to-batch reproducibility, making them suitable for compound testing and HTS. Finally, these cells can be genetically manipulated, thus allowing the stable and efficient integration and expression of genes of interest (GOIs) (Schildknecht et al., 2013; Zhang et al., 2014).

In this study, we describe the development of a LRRK2 overexpression model in LUHMES cells at early neuronal differentiation stages. We have established an easy and robust nucleofection protocol that allows stable integration of the *LRRK2* gene in proliferating LUHMES and that can be optimized for the integration of other PD-related genes. We show that LRRK2 overexpression does not interfere with normal LUHMES neuronal cell differentiation, as these cells can express DA neuronal markers and develop complex neurite networks. However, overexpression of LRRK2 induces PD-relevant phenotypic changes and these are mostly present in the G2019S line compared to the WT control. Our model can be used to interrogate LRRK2 biology and to identify novel LRRK2-mediated pathogenic mechanisms and molecular targets for disease intervention. In addition, application of this cellular model to high-throughput or high-content phenotypic screenings could greatly facilitate the discovery of new therapeutic agents for the delay or treatment of PD.

## RESULTS

### Generation and characterization of non-clonal LUHMES cells overexpressing codon-optimized WT and G2019S LRRK2

In order to validate LUHMES as a meaningful cellular system for modeling G2019S LRRK2-associated PD, we first determined whether these cells expressed endogenous LRRK2. We performed a western blot (WB) analysis for endogenous LRRK2 levels using different amounts of total protein lysate obtained from proliferating (day 0) and differentiated (day 7) naïve LUHMES cells. We observed that endogenous LRRK2 is detectable, albeit weakly, in both proliferating (Fig. S1A,B) and differentiated (Fig. S1C,D) naïve LUHMES cells, at least when using a high total protein lysate (>15 µg) and long exposure times (Fig. S1A,B). Having validated our cellular model for endogenous LRRK2 expression, we then developed a protocol for overexpressing human WT and G2019S LRRK2 in LUHMES DA-like neurons, with the aim of creating a biologically relevant, but also a rapid, scalable and cost-efficient model of PD in these cells. As classical methods (e.g. lipofection) used to introduce a GOI are hard to apply to LUHMES cells, and in order to avoid the constraints associated with the use of lentiviral vectors (Schildknecht et al., 2013), we devised an AMAXA-based nucleofection strategy to transfect LUHMES cells in the proliferating state followed by enrichment of the desired stable cell population by antibiotic selection. Validation of the nucleofection protocol was carried out using a plasmid expressing enhanced green fluorescent protein (eGFP) under the control of the *CMV* and *GAPDH* constitutive promoters. The constructs used in this study are listed in Table S1. After nucleofection, we obtained a mixed population of untransfected cells and cells with varying degrees of eGFP integration (Fig. S2A). Elimination of untransfected cells was achieved by making use of antibiotic selection (Fig. S2B). Using immunofluorescence (IF) staining, we observed that, in proliferating LUHMES, the expression of eGFP was stronger when using the *GAPDH* promoter, whereas the *CMV* promoter induced a higher eGFP expression in differentiated cells (Fig. S2B), suggesting that the two promoters had an opposite effect on transgene expression, which depended on the differentiation status of the LUHMES cells.

**Fig. 1.**
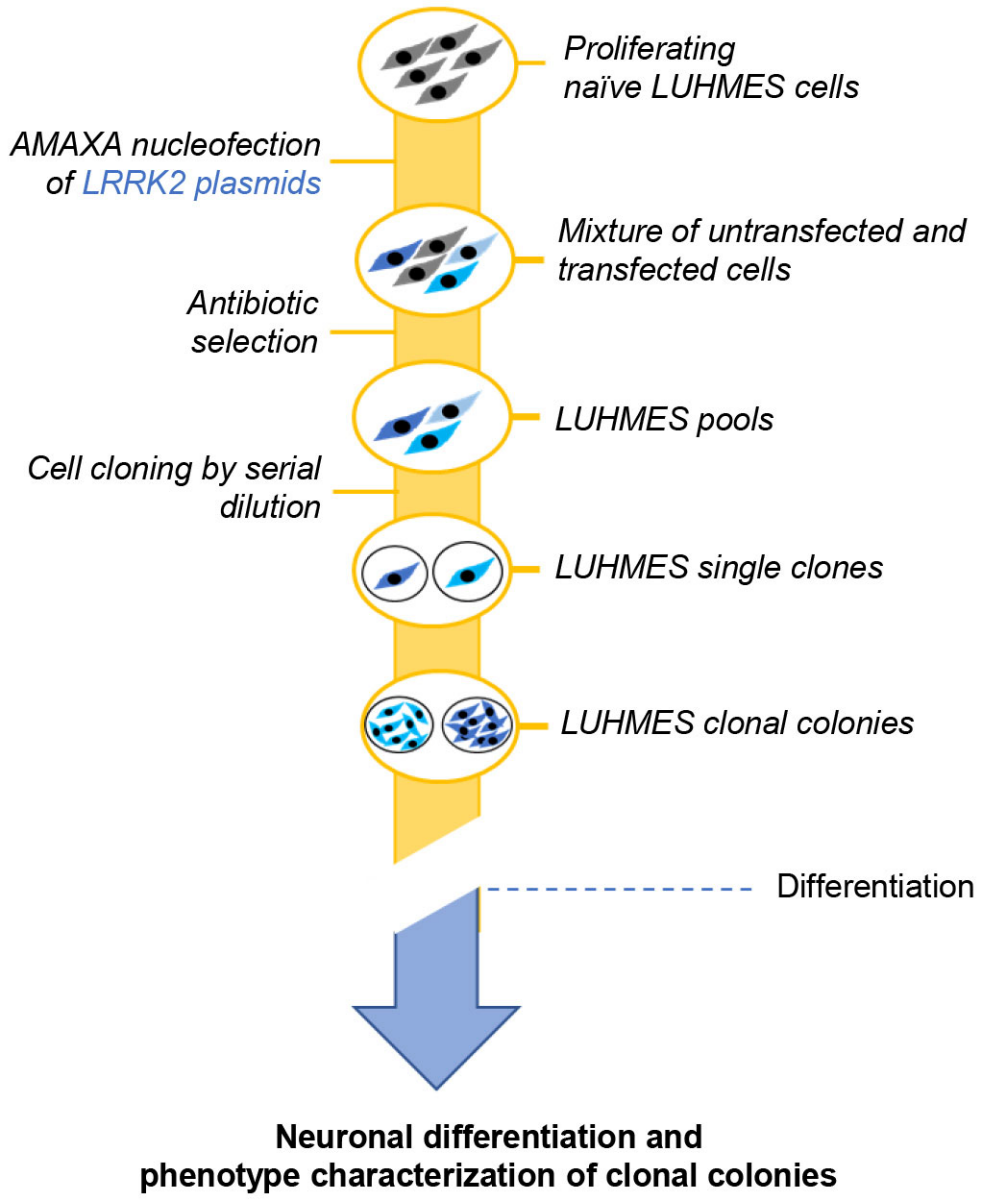
**Schematic representation of the generation of clonal LUHMES cells expressing WT and G2019S LRRK2 using AMAXA nucleofection.** Proliferating naïve LUHMES cells (gray) were transfected with plasmids expressing either WT or G2019S LRRK2 proteins (blue) using the AMAXA nucleofection technology. After transfection, a mixture of untransfected (gray) and transfected cells (blue) was obtained. Antibiotic selection removed untransfected (gray) cells, and a pool of transfected cells (LUHMES pools) expressing different levels of transgene (indicated by different shades of blue) was obtained. Serial dilution of transfected pool generated single clones that subsequently formed clonal colonies. LUHMES cell pools and clonal colonies were differentiated into mature neurons and further characterized as described in the main text.

Having established that our nucleofection strategy could be effectively used to introduce genetic material in proliferating LUHMES cells, we then applied this protocol to separately transfect plasmids expressing our two GOIs, the human WT and G2019S *LRRK2*, under the control of the same promoters used for eGFP expression. A schematic representation of the workflow used to generate and characterize LUHMES cells overexpressing LRRK2 is shown in Fig. 1. The choice of using two different constitutive promoters was made with the goal of increasing the probability of driving expression of the *LRRK2* gene in LUHMES cells. Using WB analysis, we observed no expression of the transgenic non-codon optimized WT and G2019S LRRK2 proteins in the transfected cell population (Fig. S3A-D). Several factors can account for weak transgenic *LRRK2* expression in cells, such as the length of the transcribed protein, the amino acid composition of the leucine-rich repeat, and the presence of rare codons. As we found that the *LRRK2* sequence carries several rare codons, we decided to use a codon-optimized version of the *LRRK2* DNA (Table S2) to maximize the likelihood of higher protein expression in our cellular context. By doing so, we were able to successfully obtain a robust LRRK2 expression in LUHMES cells, as observed in WB experiments (Fig. S3A-D). We also observed that the *CMV* promoter led to a higher expression of codon-optimized LRRK2 compared to the *GAPDH* promoter in differentiated LUHMES cells (Fig. S3A-D).

Selection of transfected LUHMES cells was obtained using puromycin. We refer to this non-clonal cell population as the LUHMES pool. For further characterization of the WT and G2019S LRRK2 LUHMES pools, the levels of LRRK2 were analyzed by WB during the LUHMES differentiation course (Fig. S4A). In naïve LUHMES, endogenous LRRK2 expression was not detected in proliferating or in differentiating cells in the experimental conditions used (total protein lysate equal to 10 µg and exposure time of 60 s). Expression of the transgenes was robustly achieved in the LUHMES pools and the highest expression was reached around day 4 of differentiation for both promoters (Fig. S4A). Analogously to what was observed for the eGFP protein (Fig. S2), expression of LRRK2 in the proliferating state (day 0) was favored by the *GAPDH* promoter, whereas the *CMV* promoter favored LRRK2 expression in differentiating LUHMES (Fig. S4A). A decrease in LRRK2 levels was observed at later differentiation times (days 7 and 11; Fig. S4A), with LRRK2 G2019S being considerably more affected.

Having determined that we could obtain robust LRRK2 levels in the LUHMES pools, we then analyzed the effect of LRRK2 expression on the ability of LUHMES cells to correctly differentiate into DA-like neurons. For this, we used WB and IF techniques to analyze the expression of neuronal markers such as SNCA, tyrosine hydroxylase (TH), βIII-tubulin (TUJ1) and microtubule-associated protein 2 (MAP2) at different times of differentiation (Figs. S4B-D and S5A) and compared them to naïve LUHMES cells. Expression levels of all the markers increased with the differentiation time, and their kinetics profiles were comparable between naïve and transgenic cells. Although TUJ1 was already present in proliferating cells (Fig. S4D) and reached a plateau around day 4 (Fig. S4D), TH and MAP2 became visible only at later differentiation times (Figs. S4B-D and S5A), in agreement with previously reported data (Lotharius et al., 2002). Weak SNCA expression was visible by immunocytochemistry (ICC) already at differentiation day 1 (Fig. S5A), but was not observed in the WB analysis (Fig. S4B) due to the experimental conditions used in this experiment (10 µg total protein lysate and low exposure time). In addition, SNCA expression was mainly found in the cell bodies at the short exposure time chosen for the IF data (Fig. S5A), but we confirmed SNCA expression in neurites when using a different antibody (Fig. S5B). Of interest, we observed that expression of the TH marker was negatively affected by the expression of the mutant LRRK2 protein at all differentiation time points tested (Figs. S4C and S5A,C). We quantified the TH^+^ cells in WT and G2019S (GS) LRRK2 LUHMES pools and compared them to the number of TH^+^ cells in naïve LUHMES cultures. Whereas TH^+^ cell numbers were similar in naïve and LUHMES cells overexpressing WT LRRK2 (Fig. S5C), overexpression of the G2019S LRRK2 protein caused a significant reduction in TH^+^ cells (Fig. S5C), in agreement with the WB results (Fig. S4C) and with previous reports (Borgs et al., 2016). Nonetheless, both types of cell lines were able to generate a high number of DA-like neurons, allowing us to study their early differentiation steps.

### Generation of clonal LUHMES cells overexpressing WT and G2019S LRRK2

**Fig. 2.**
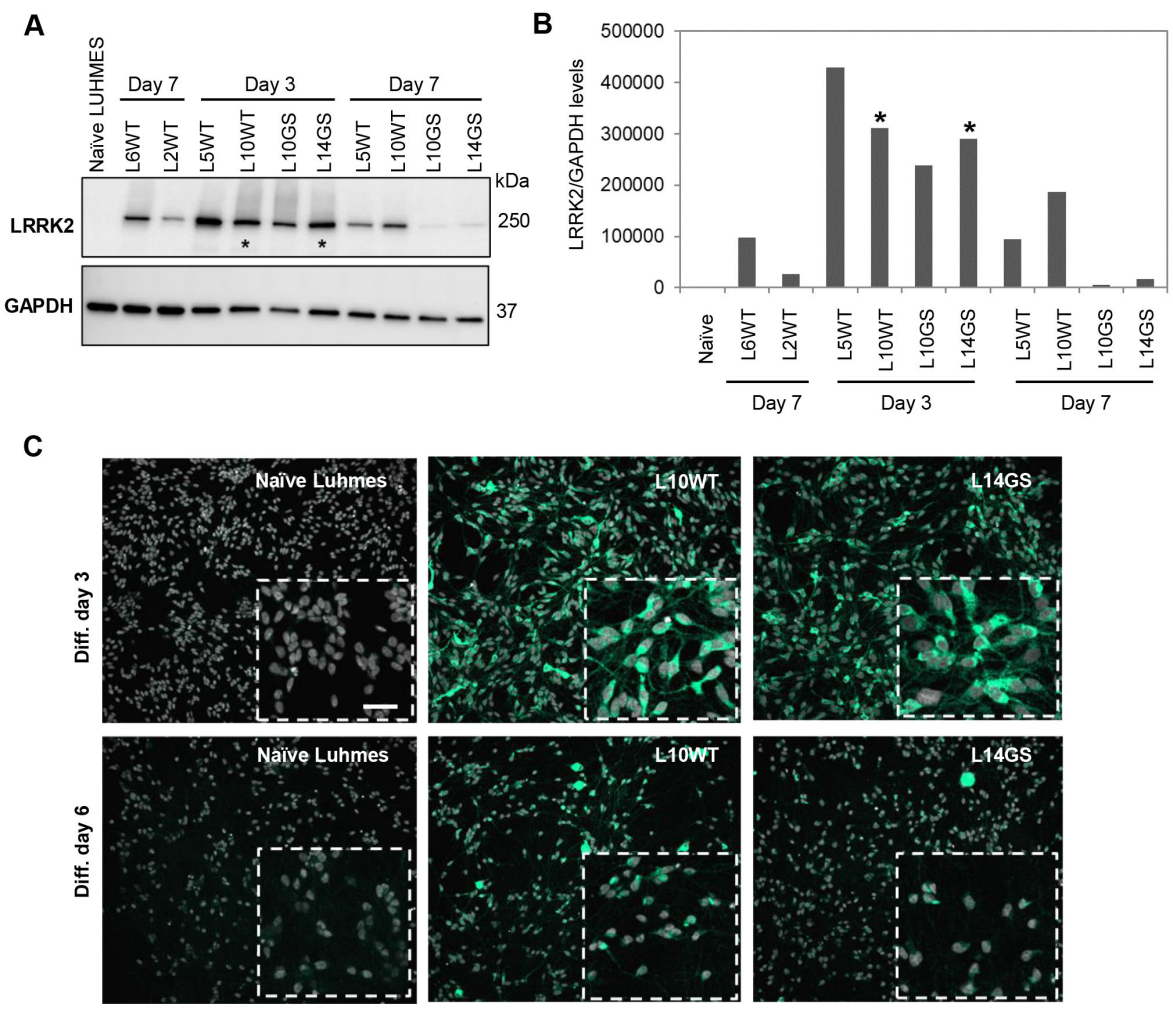
**LUHMES cell clones express WT and G2019S LRRK2 proteins.** (A) Western blot (WB) analysis of LRRK2 levels in naïve LUHMES cells and in different wild-type (WT; L6WT, L2WT, L5WT and L10WT) and G2019S (L10GS, L14GS) clones at days 3 and 7 of LUHMES cell differentiation. L10WT and L14GS clones expressed similar levels of LRRK2 at day 3 of LUHMES cell differentiation (indicated by asterisks). At a later differentiation time (day 7), the expression levels of LRRK2 decreased, with the mutant protein being more drastically affected. The housekeeping gene *GAPDH* was used as a loading control. The displayed experiment is representative of three independent experiments. (B) WB quantification of LRRK2 levels normalized to GAPDH. Asterisks denote clones with equivalent LRRK2 levels. (C) Immunofluorescence (IF) analysis of LRRK2 levels in naïve, L10WT and L14GS LUHMES clones at days 3 and 6 of LUHMES cell differentiation. Endogenous LRRK2 levels are close to background noise in naïve LUHMES cells. However, transgenic LRRK2 is clearly visible in the two clones, especially at day 3 of differentiation. Analogously to the WB results in A, LRRK2 levels decrease at day 6 of differentiation and this decrease is more evident in the mutant L14GS clone. LRRK2 is shown in green; nuclei are shown without pseudocolor. For direct comparison, images were taken with the same exposure time. The displayed experiment is representative of three independent experiments. Scale bar: 50 µm.

As the LRRK2 transfected pools differentiated similarly to naïve LUHMES cells, we then went on to make single-cell clones in order to obtain homogeneous cultures (Fig. 1). LUHMES cells do not survive when grown in culture at low cell density. Therefore, in a first step to obtain single-cell colonies, we made serial dilutions of antibiotic-selected cultures in six-well plates and picked the colonies derived from single cells before applying a second round of antibiotic selection. Using WB and IF techniques, we demonstrated that the antibiotic-resistant colonies grown from these single clones were all successfully expressing the LRRK2 proteins, despite showing a different level of expression, most probably due to the number of constructs incorporated in a single cell and to the efficiency of transcription, which depends on the genome integration site (Fig. 2A-C; Fig. S6A,B). As observed for the LUHMES pools, all transgenic clones expressed LRRK2 at a significantly higher level than the naïve LUHMES cells, for which LRRK2 expression was not detectable by WB at the conditions used for the detection of LRRK2 in overexpressing LUHMES pools (Fig. 2A,B; Fig. S6A) and by ICC (Fig. 2C; Fig. S6B). In general, we observed that, when compared to naïve LUHMES cells at the same stage of differentiation, transgenic cells expressed the WT LRRK2 protein at a higher level than the cells expressing the G2019S protein (Fig. 2A-C; Fig. S6A). As expected, overexpression of G2019S LRRK2 led to higher phosphorylation of the Ser1292 residue than overexpression of WT LRRK2 (Fig. S6C,D), and this effect was dependent on the mutant protein as the phosphorylation band disappeared in its absence (Fig. S6C,D). We also observed that the levels of LRRK2 decreased at about days 6-7 of LUHMES differentiation and that the decreased LRRK2 levels were more pronounced for the mutant protein (Fig. 2A-C; Fig. S6A-C). Although *LRRK2* mRNA levels (Fig. S7A,B) inversely correlated with the WB results (Fig. 2A) for the same day of differentiation, when comparing the mRNA and protein levels of WT and G2019S LRRK2 at the same time point, the results of quantitative PCR (qPCR) analysis reflected the trend observed by WB and suggested that the reduction in G2019S LRRK2 protein levels during LUHMES cell differentiation could be due to gene silencing (Fig. S7B). Taken together, these results suggested that sustained overexpression of the LRRK2 protein may be not tolerated by differentiated post-mitotic LUHMES cells, and that expression of the mutant protein may be more negatively impacted than its WT counterpart. This result is not surprising, considering that DA neurons do not normally express high levels of LRRK2 (Galter et al., 2006; Han et al., 2008; Higashi et al., 2007; Mandemakers et al., 2012; Melrose et al., 2006).

### Characterization of clonal LUHMES cells overexpressing WT and G2019S LRRK2

**Fig. 3.**
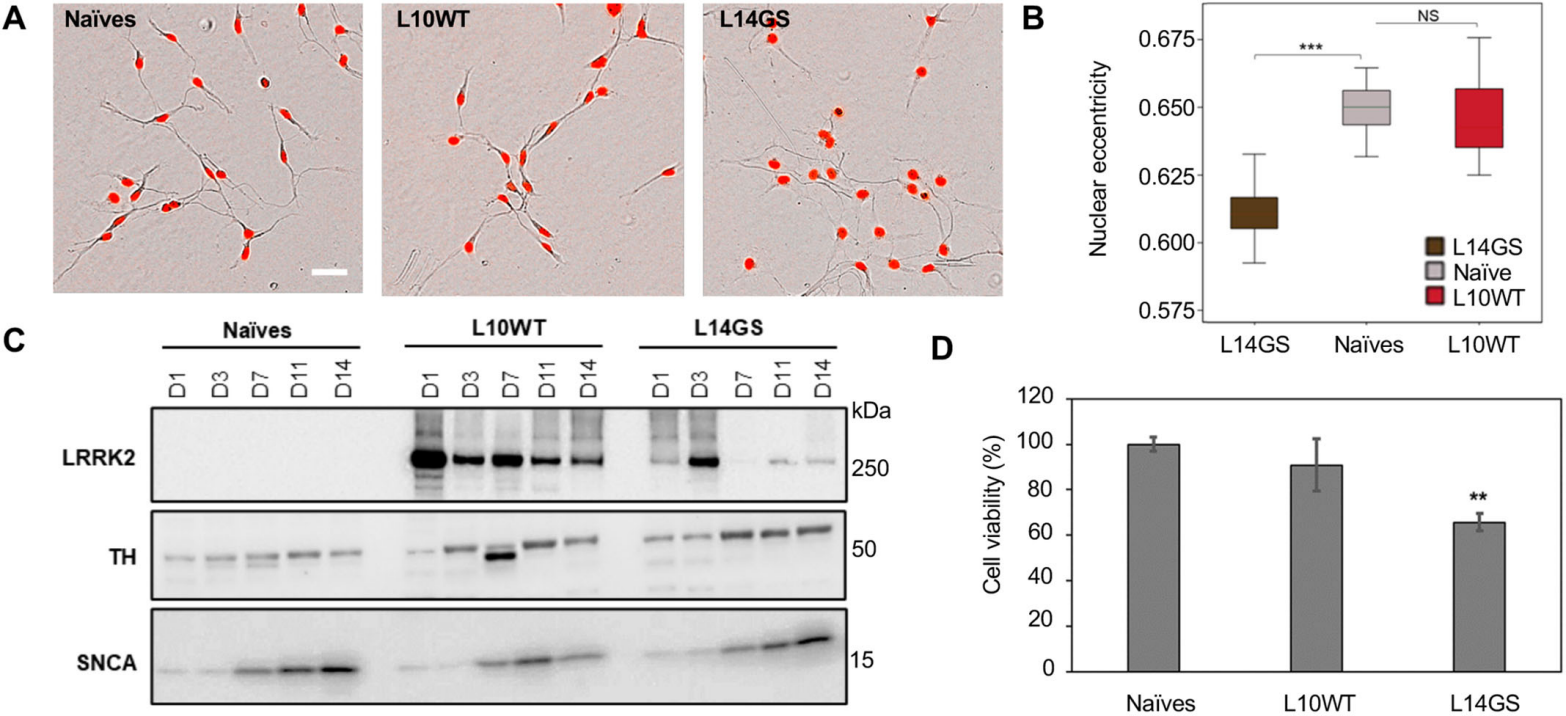
**LUHMES cell clones differentiate into neurons and express dopaminergic markers.** (A) Morphological analysis of naïve and LRRK2-expressing LUHMES clones (L10WT and L14GS) was performed in live cells using the Incucyte S3 analysis system. Several parameters were analyzed with the Incucyte Neurotracker software using brightfield images. Nuclei were stained with the NucLight reagent (red). The displayed experiment is representative of two independent experiments. Scale bar: 25 µm. (B) Differences in nuclear shape (eccentricity) were statistically significant between the G2019S clone L14GS and the naïve LUHMES cells. WT LUHMES clone (L10WT) behaved similarly to the naïve cells. Error bars show mean±s.d. One-way ANOVA with Tukey's post-hoc test was performed. Differences with *P*≤0.05 were considered significant, with *n*≥10 from two independent experiments. ***P<0.001; NS, not significant. (C) LRRK2 levels together with dopaminergic markers such as tyrosine hydroxylase (TH) and α-synuclein (SNCA) were analyzed by WB during LUHMES cell differentiation [day 1 (D1) to day 14 (D14)]. LRRK2 overexpression in LUHMES clones did not interfere with neuronal maturation and differentiation. Expression of TH and SNCA was not affected by LRRK2 overexpression. Both markers followed a kinetic analogous to that of naïve cells (at least for the WT clone, for which LRRK2 expression was relatively stable until day 14). Protein level was normalized by total protein load using Stain-Free technology. The displayed experiment is representative of two independent experiments. A total protein lysate of 20 µg was loaded on the gel, and for imaging an exposure time of 60 s was used. The protein corresponding molecular mass (in kDa) is indicated on the right side of the panel. (D) LUHMES cell viability was measured using the Cell TiterGlo viability assay. A 30% increased cell toxicity was found in the L14GS clone. Results are expressed as mean percentage of viability compared to naïve LUHMES cells (100% viability); sample size *n*≥8 per cell line; experiments performed in triplicate. Error bars show mean±s.d. One-way ANOVA with Tukey's post-hoc test was performed. **P<0.01.

Our goal was to develop a rapid cellular assay with broad potential for being used in basic research, to be scalable and relatively inexpensive for HTS. Consequently, we decided to focus our studies on the LUHMES clones in which LRRK2 expression was under the control of the *CMV* promoter, as it ensured a higher level of LRRK2 protein in differentiated cells as compared to the *GAPDH* promoter (Fig. S4A). We hypothesized that the high LRRK2 levels, despite not mimicking physiological conditions in DA neurons, would nevertheless produce a useful system for exploring LRRK2 biology as it would drive LRRK2 phenotypes at early time points. Using WB analysis, we identified two independent sets of clonal lines of the LRRK2 variants with comparable transgene expression: L10WT and L14GS clones (Fig. 2A) together with the L6WT and L2GS clones (Fig. S6A). Consequently, these clones were used in multiple sets of experiments, with most experiments performed on clones L10WT and L14GS. In order to determine whether LRRK2 expression would compromise the differentiation of clonal LUHMES cells into DA-like neurons, we verified the cell morphology (Fig. 3A,B) and the expression of neuronal markers such as TH and SNCA at different differentiation time points (Fig. 3C). No gross abnormalities in cell shape, size or neurite length were detected, although a loss of the nuclear eccentricity was consistently seen in G2019S-expressing cells (Fig. 3A,B). Nuclei in G2019S LRRK2-expressing cells were more circular and appeared larger in size compared to naïve and WT LUHMES cells. Just as the WT and G2019S clones, L10WT and G14GS, express equivalent LRRK2 levels, this phenotype is attributable to the increased kinase activity of the mutant protein, in accordance with a recent study (Chen et al., 2020).

In agreement with the majority of the morphological readouts, the levels of the neuronal markers were also not altered by the overexpression of LRRK2 (Fig. 3C). In fact, both TH and SNCA followed the expression kinetic of naïve LUHMES: the two markers were present from the start of the differentiation (day 1) and their levels increased with the differentiation time reaching a plateau around day 11, despite TH expression being slightly affected by the G2019S LRRK2 mutant at differentiation day 3 (Fig. 3C).

A pathological hallmark of PD is the robust degeneration of substantia nigra DA neurons (Dickson, 2012). Therefore, we investigated whether expression of the mutant LRRK2 protein would cause DA cell death. WT LRRK2 expression in the L10WT clone did not induce any significant neuronal loss (Fig. 3D). In contrast, under the same conditions and levels of LRRK2 expression, the G2019S LRRK2 mutant caused a progressive degeneration of DA neurons (Fig. 3D), suggesting that the observed toxicity was dependent on the G2019S mutation. These results agree with the findings by Dusonchet et al. (2011), which showed a progressive degeneration of DA neurons mediated by mutant G2019S LRRK2 overexpression in a rat model of PD. Taken together, our results suggest that the differentiation ability of the LUHMES cells and their general phenotype was not impacted by LRRK2 overexpression, at least at early time points before LRRK2 disappearance. However, LRRK2 G2019S impacted the viability of LUHMES cells already at these early differentiation time points.

**Fig. 4.**
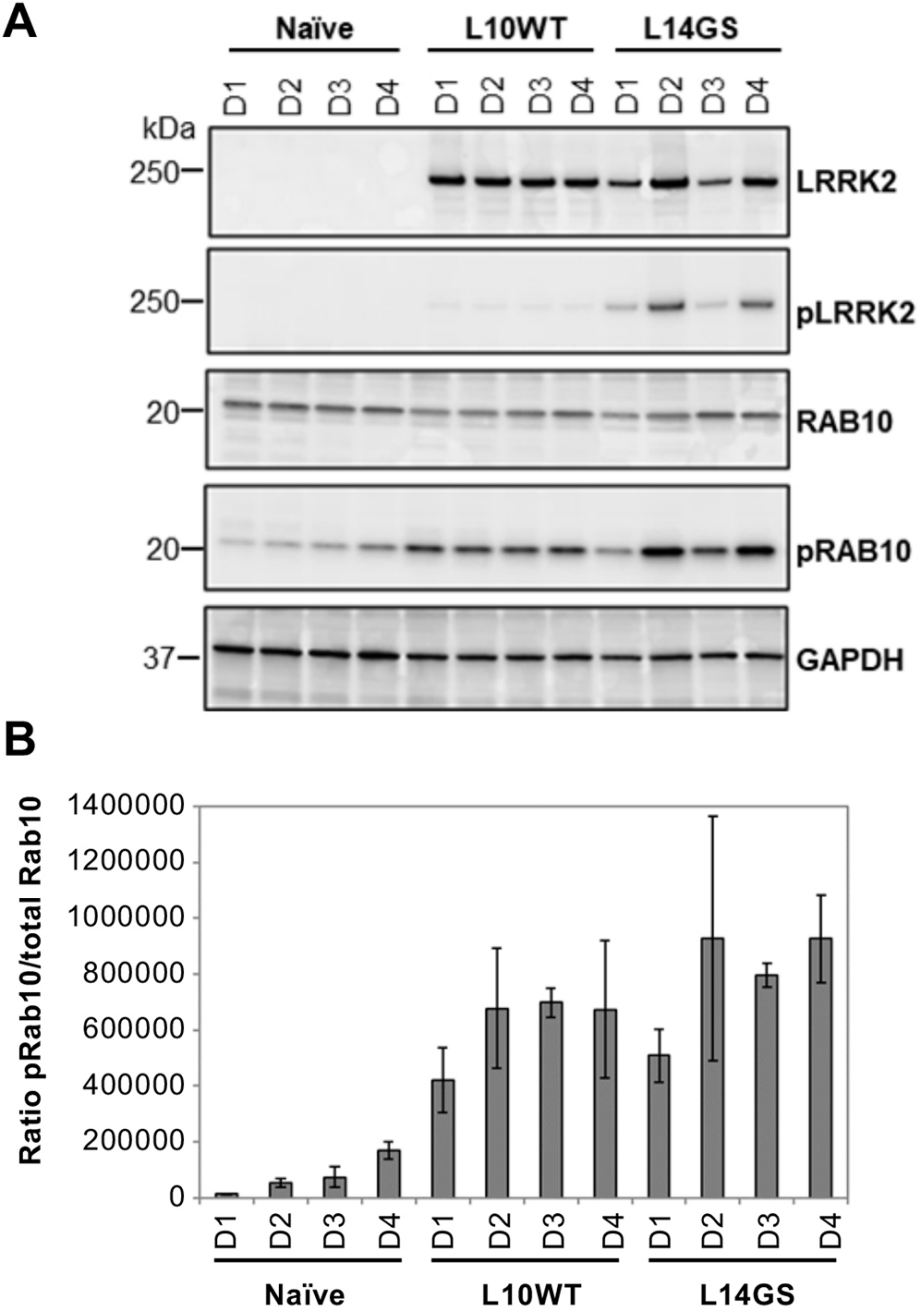
**Phosphorylation of bona fide LRRK2 substrates in LUHMES clones.** (A) Representative WB results showing the phosphorylation of two LRRK2 bona fide substrates, LRRK2 Ser1292 and RAB10 Thr73, in naïve and clonal LUHMES cells (L10WT and L14GS). LUHMES cells were differentiated for up to 4 days, and levels of total and phosphorylated LRRK2 and RAB10 were analyzed each day. GAPDH was used to ensure equal loading. The protein corresponding molecular mass (in kDa) is indicated on the left side of the panel. (B) Quantification of pRAB10 levels. Results from six independent experiments. Error bars show mean±s.d.

Pathogenic LRRK2 mutations reproducibly increase phosphorylation of two well-established LRRK2 physiological substrates both *in vitro* and *in vivo*: the Ser1292 LRRK2 autophosphorylation site (Sheng et al., 2012) and the Rab GTPase family members (Steger et al., 2016) as heterologous substrates. To monitor LRRK2 kinase activity, we investigated whether the overexpression of WT and G2019S LRRK2 would increase the phosphorylation of these two established LRRK2 substrates compared to naïve cells. LUHMES cells were differentiated for up to 4 days and levels of total and phosphorylated LRRK2 and RAB10 were analyzed by WB at each differentiation day (Fig. 4). We expected that LRRK2 overexpression would lead to a significant increase in substrate phosphorylation compared to the naïve LUHMES, for which LRRK2 expression is undetectable. As anticipated, naïve LUHMES did not show any Ser1292 phosphorylation, but displayed an increase in phosphorylated (p)RAB10 levels with the cell differentiation time (Fig. 4). Overexpression of WT LRRK2 led to a weak increase in pSer1292 level, which was an unexpected result considering that the levels of WT LRRK2 were much higher than those present in the naïve LUHMES. RAB10 phosphorylation levels were instead elevated in WT LRRK2 samples and reached a plateau by days 2-3 of differentiation (Fig. 4). Only the expression of G2019S LRRK2 led to a considerable increase in both Ser1292 and RAB10 phosphorylation levels (Fig. 4), and this increase was due to the mutant higher catalytic activity as the total levels of WT and G2019S LRRK2 were comparable.

### Treatment of LUHMES cells overexpressing WT and G2019S LRRK2 with the LRRK2 inhibitor MLI-2

**Fig. 5.**
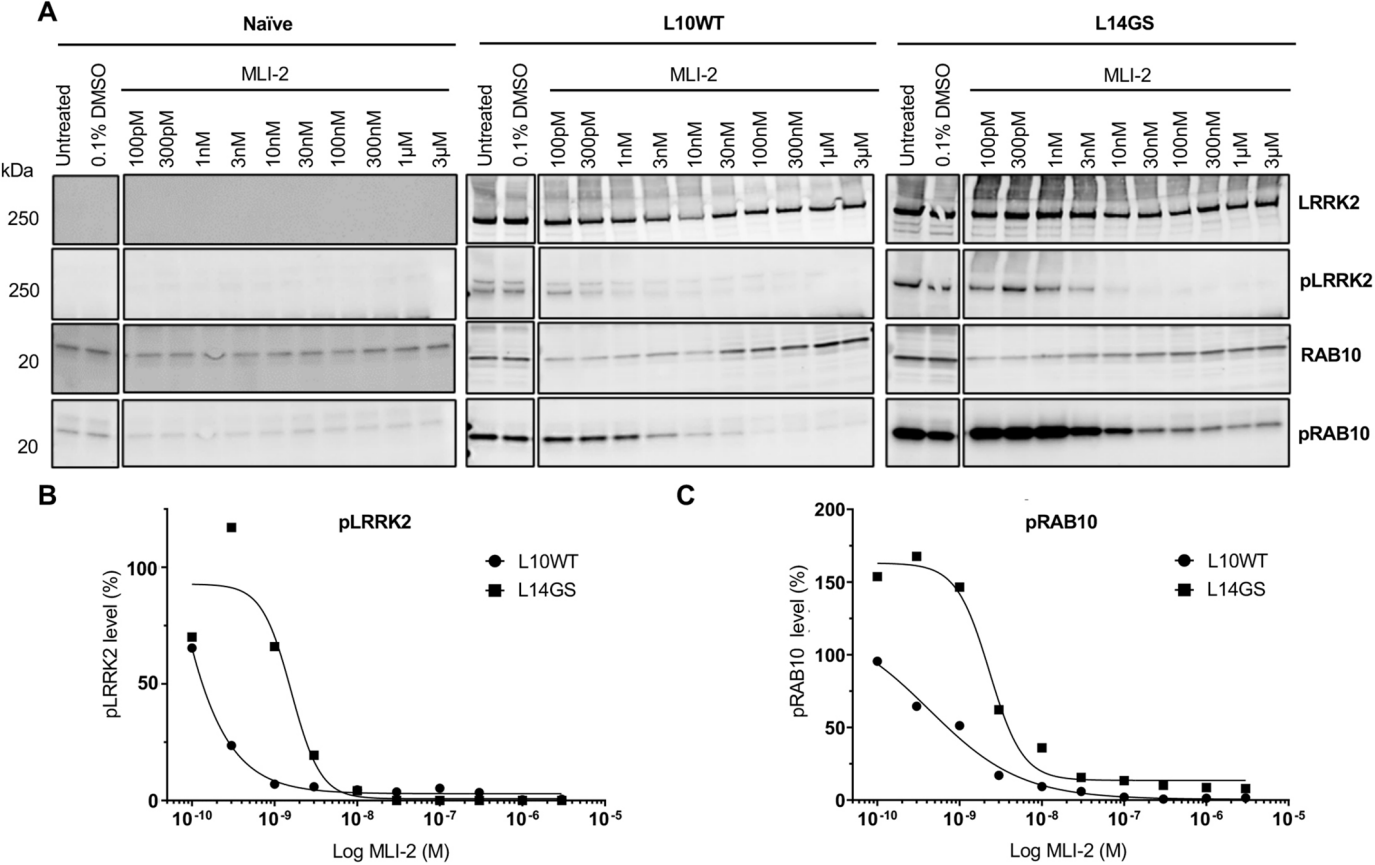
**LRRK2 kinase inhibitor MLI-2 effectively reduces phosphorylation of two bona fide** **LRRK2** **substrates.** (A) LUHMES cells were differentiated for 2 days and then treated with increasing concentrations of LRRK2 kinase inhibitor MLI-2 for 4 h. A potent reduction in the phosphorylation of LRRK2 Ser1292 and RAB10 Thr73 was observed. (B,C) Dose-response curves of the LRRK2 kinase inhibitor MLI-2 against LRRK2 pSer1292 and RAB10 pThr73 in WT and G2019S LUHMES cells. The protein corresponding molecular mass (in kDa) is indicated on the left side of the panel. (B) Half-maximal inhibitory concentration (IC_50_) values, calculated for WT and G2019S LRRK2 by GraphPad Prism software on LRRK2 pSer1292, were found to be 21.2 pM (L10WT clone, filled circles) and 1.45 nM (L14GS clone, filled squares). (C) IC_50_ values, calculated by GraphPad Prism software for WT and G2019S LRRK2 on RAB10 pThr73, were found to be 0.78 nM (L10WT clone, filled circles) and 2.31 nM (L14GS clone, filled squares). Results from three independent experiments.

Potent and selective LRRK2 kinase inhibitors have been developed (Choi et al., 2012; Deng et al., 2011; Fell et al., 2015; Henderson et al., 2015; Ramsden et al., 2011; Reith et al., 2012; Thirstrup et al., 2017), and have been shown to reduce phosphorylation at Ser935/Ser1292 (Atashrazm et al., 2019; Fell et al., 2015; Ito et al., 2016; Lobbestael et al., 2016) and Thr73 of the LRRK2 and RAB10 proteins (Atashrazm et al., 2019; Thirstrup et al., 2017), respectively. Consequently, a widely used readout for LRRK2 kinase inhibition in cellular contexts is the dephosphorylation of these above-mentioned LRRK2 substrates. As Ser935 is not exclusively phosphorylated by LRRK2 (Sheng et al., 2012), we decided to focus our studies on the autophosphorylation site Ser1292, in addition to investigating the effects of LRRK2 inhibition on residue Thr73 of the RAB10 protein. To investigate the effect of pharmacological LRRK2 kinase inhibition in our cellular model, LUHMES clones with and without LRRK2 overexpression were treated with different concentrations of the kinase inhibitors MLI-2 (Fell et al., 2015; Scott et al., 2017), and the phosphorylation levels of Ser1292 and Thr73 were examined by WB. As expected, in untreated and DMSO-treated cells, an increase in substrate phosphorylation was correlated with the degree of LRRK2 kinase activity (G2019S LRRK2>WT LRRK2>endogenous LRRK2; Fig. 5A). LRRK2 G2019S, in fact, gave the highest increase in phosphorylation for both LRRK2 pSer1292 and RAB10 pThr73, followed by WT LRRK2, which in turn led to a higher substrate phosphorylation than endogenous LRRK2 in naïve cells. Treatment of cells with increasing concentrations of the potent LRRK2 kinase inhibitor MLI-2 induced a rapid dephosphorylation of both LRRK2 pSer1292 and RAB10 pThr73 substrates (Fig. 5A). This effect was rapid and strong, as we detected a decrease in phosphorylation after only 4 h of compound incubation with the cells. MLI-2 inhibited substrate phosphorylation in a dose-dependent manner, with half-maximal inhibitory concentration (IC_50_) values for the inhibitor of 21.2 pM and 1.45 nM for pSer1292 in WT and G2019S LRRK2, respectively, and of 0.78 nM and 2.31 nM for RAB10 pThr73 in WT and G2019S LRRK2, respectively, in agreement with data reported previously, albeit in a different cellular context (Fig. 5B,C) (Di Maio et al., 2018; Ito et al., 2016).

### Mitochondrial phenotypes driven by mutant LRRK2 expression

Multiple studies implicate LRRK2 in mitochondrial dynamics, function and quality control (reviewed in Singh et al., 2019). LRRK2 has been shown to colocalize to mitochondria and interact with a number of key regulators of mitochondrial fission/fusion (Stafa et al., 2014; Wang et al., 2012), thus raising the possibility that LRRK2 may lead to PD pathogenesis by disrupting mitochondria function when overexpressed or mutated (Biskup et al., 2006). Mitochondrial impairment has been observed in post-mortem human tissues from PD patients with LRRK2 mutations, various animal models of G2019S LRRK2-mediated PD (Cooper et al., 2012; Hsieh et al., 2016; Mortiboys et al., 2010; Sanders et al., 2014; Yue et al., 2015) and cellular models of the disease (Cherra et al., 2013; Niu et al., 2012; Singh et al., 2019; Su and Qi, 2013; Wang et al., 2012).

**Fig. 6.**
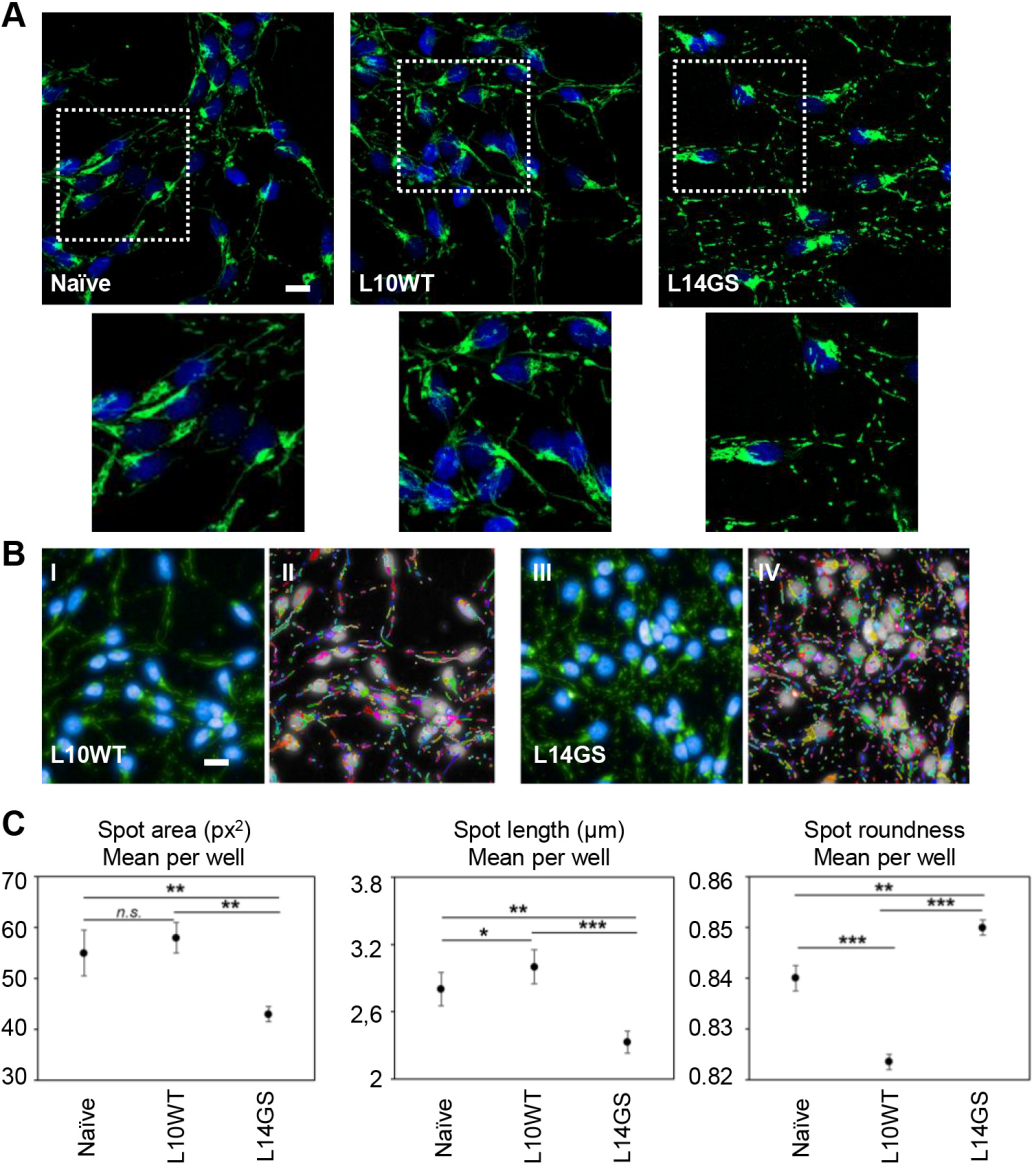
**Effect of LRRK2 overexpression on mitochondria morphology in LUHMES cells.** (A) Naïve, WT (L10WT) and G2019S (L14GS) LUHMES clones were differentiated for 3 days and then fixed and immunostained with the anti-TOM20 antibody to identify mitochondria. Green, TOM20; blue, Hoechst 33342. Insets show enlargements of boxed areas. Fragmented mitochondria are clearly visible in the G2019S (L14GS) clone. Scale bar: 10 µm. The displayed experiment is representative of three independent experiments. (B) Representative pre-processed IF images (I,III) of WT and G2019S LUHMES clones showing mitochondria morphology (green, TOM20; blue, Hoechst 33342) and segmentation (II,IV). Scale bar: 10 µm. (C) Quantification of mitochondrial morphology in L14GS clone revealed a significant decrease in the aspect ratio (left and center), and an increase in the percentage of cells displaying rounder mitochondria (right) compared to naïve and L10WT LUHMES. Experiments were repeated three times. Data are presented as mean±s.d. *n*≥6 per cell line. Statistical significance was examined by ANOVA and Tukey's multiple comparisons test with a statistical criterion of 0.05. *P<0.05, **P<0.01, ***P<0.001; n.s., not significant.

As a multitude of studies has shown that LRRK2 mutants can alter mitochondrial fusion/fission balance (Grunewald et al., 2014; Niu et al., 2012; Smith et al., 2016; Su and Qi, 2013; Wang et al., 2012), we investigated the effect of overexpressing WT and G2019S LRRK2 on mitochondrial morphology in differentiated LUHMES cells. For this purpose, the two pairs of LUHMES clones expressing similar levels of WT and G2019S LRRK2 (L6WT, L10WT, L10GS and L14GS) were differentiated for up to 3 days and then fixed, and mitochondria were identified by staining with two different markers: the antibody for the mitochondrial import receptor subunit TOM20 (Fig. 6A) and the probe MitoTracker Green (Fig. S8A). In undifferentiated cells, mitochondria were all clumped around the nuclei and no difference was noticeable between the WT and mutant LRRK2-expressing cells (Fig. S8B). In contrast, after 3 days of LUHMES differentiation, we observed that the mitochondria morphology was deeply affected by G2019S LRRK2 expression (Fig. 6A). In fact, whereas mitochondria shape was elongated in the neurites of naïve LUHMES cells and in cells expressing WT LRRK2, these organelles appeared fragmented in the G2019S-expressing clones (Fig. 6A). Moreover, we observed an increase in mitochondrial content mainly in the perinuclear area of the mutant cells. These results were subsequently confirmed by staining the mitochondria with the probe MitoTracker Green (Fig. S8).

In order to exclude any potential bias and with the final goal of developing this cellular model for future HTS applications, we devised an algorithm for automatically assessing mitochondria morphology and number using the Harmony analysis software available in the Operetta CLS high-content imaging system (Fig. 6B). After wells were scanned, mitochondria number was normalized to the number of nuclei/well. Mitochondrial morphology was assessed using the mitochondrial import receptor subunit TOM20 (also known as TOMM20) antibody. This assay utilized two additional dyes to enable the identification of individual cells by automated image analysis: Hoechst 33342 was used to identify nuclei (Fig. 6B) and wheat germ agglutinin (WGA) was used to identify the cell soma (Fig. S8C). Mitochondrial objects clumped around the nuclei were eliminated by size exclusion and only mitochondria in the neurites were used for the analysis. These mitochondria were then associated with their related cell soma and the number of objects per cell was counted. Three measures of morphology were assessed: the area of each mitochondrion identified (averaged per cell number and then per well), the major axis length of each mitochondrion identified within a cell (averaged per cell and then per well), and the roundness (the width divided by the length) of mitochondria. The mean area of mitochondria, the major axis length and the roundness are measures that indicate how interconnected or fragmented the mitochondria are. In general, mitochondria in naïve LUHMES cells presented a morphology more similar to those of the L10WT clone, even if statistical analysis showed a significant difference in the spot length and roundness of the mitochondria between these two lines (Fig. 6C, middle and right), indicating that the mitochondria in the naïve cells were a little shorter than the ones in the L10WT clone. Nonetheless, the size of mitochondria in the L14GS clone was always dramatically smaller than that of the naïve LUHMES and L10WT clone (Fig. 6C). In G2019S cells, mitochondria appeared smaller and rounder compared to the mitochondria of naïve and WT cells (Fig. 6C). Reduced average area and length of mitochondria suggest that the mitochondria of G2019S LRRK2-expressing cells were more fragmented, which is consistent with previous reports using other models (Ho et al., 2018; Niu et al., 2012; Su and Qi, 2013; Walter et al., 2019).

### Lysosomal phenotypes driven by mutant LRRK2 expression

A hallmark of PD is the accumulation of proteinaceous inclusions rich in SNCA, named Lewy bodies (Dauer and Przedborski, 2003; Wakabayashi et al., 2007), which suggests impaired protein clearance as a contributor of disease pathogenesis. Supporting this observation is the reduction in lysosomal markers and increase in accumulation of autophagosomes in the post-mortem brain samples of PD patients (Arotcarena et al., 2019). In addition, genetic data showing that several genes linked to PD – such as *SNCA*, *GBA*, *VPS35* and *LRRK2* – converge on the lysosomal pathway, reinforce the link between PD and lysosomal impairment (Smolders and Van Broeckhoven, 2020). Multiple studies suggest that LRRK2 alters lysosome morphology (Henry et al., 2015; Hockey et al., 2015; MacLeod et al., 2006; Schapansky et al., 2018); therefore, we explored the effect of overexpressing WT and G2019S LRRK2 on lysosomal phenotype in LUHMES cells.

**Fig. 7.**
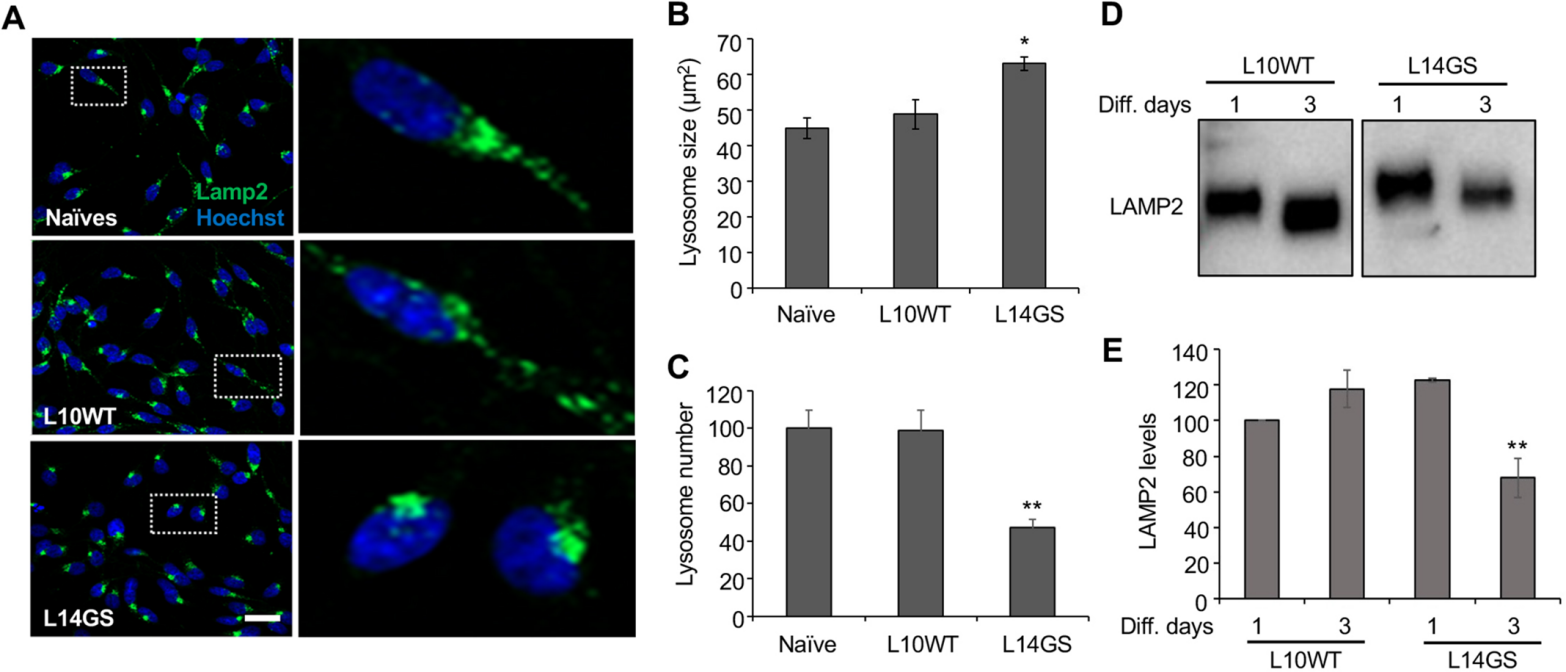
**Overexpression of G2019S LRRK2 leads to enlarged, perinuclear lysosomes in LUHMES cells.** (A) The L10WT and the L14GS clones were differentiated for 3 days and then fixed, permeabilized and stained with the anti-LAMP2 antibody to identify lysosomes. Green, LAMP2; blue, Hoechst 33342. Enlargements of boxed areas are shown on the right. G2019S (GS) clone L14GS presents enlarged and perinuclear lysosomes compared to the WT clone L10WT. Scale bar: 25 µm. The displayed experiment is representative of two independent experiments. (B,C) An algorithm was generated to determine lysosome size and number in LUHMES cells. At least three wells per line were analyzed, and lysosome size and number were normalized to the number of cells per well. The result is representative of three independent experiments. (D) LAMP2 levels were analyzed at days 1 and 3 of differentiation. At day 3, the levels of LAMP2 in the G2019S clone are reduced compared to the WT clone. Results are representative of two independent experiments. (E) Quantification of LAMP2 WB results. Densitometric analysis of LAMP2 levels normalized by total protein load using Stain-Free technology. Results are representative of two independent experiments. For experiments in B, C and E, data are presented as mean±s.d. Statistical significance was examined by one-way ANOVA with Tukey's multiple comparison test. *n*≥6 from two independent experiments. *P<0.05, **P<0.01.

We began our studies by looking for any major differences in lysosome morphology between cells overexpressing WT and G2019S LRRK2. We used the lysosomal membrane protein LAMP2 antibody to identify lysosomes and co-stained the cells with the nuclear dye Hoechst 33342 to count the number of cells per field and to distinguish perinuclear from distal lysosomes. Algorithms were generated to measure average lysosome size and number. Results revealed a higher mean lysosomal area and an increased lysosome clustering around the nuclei (Fig. 7A) specifically in LRRK2 G2019S cells, in agreement with data obtained in human fibroblasts (Hockey et al., 2015), in primary mouse astrocytes (Henry et al., 2015) and in follicle cells of *Drosophila* (Dodson et al., 2012), but in contrast to the findings obtained in knock-in mouse neurons (Schapansky et al., 2018). Lysosomes in LRRK2 G2019S cells were significantly larger compared with LUHMES cells expressing equivalent level of WT protein, demonstrating that the effect on lysosome size was not due to LRRK2 G2019S overexpression, but to the mutation itself (Fig. 7A,B). The number of lysosomes per cell was also significantly reduced in LUHMES cells overexpressing the mutant protein (Fig. 7C). The decrease in lysosome number in the mutant line was paralleled by changes in the levels of the LAMP2 marker, as detected by WB analysis (Fig. 7D,E). Altogether, these results indicate that LRRK2 G2019S causes an increase in lysosomal size and an alteration in lysosome localization, thus probably affecting the lysosomal function and capacity of the cell.

## DISCUSSION

Cellular models of PD are valuable tools for the understanding of PD pathogenesis and for drug discovery because they can contribute to elucidating disease pathological mechanisms while being amenable to HTS of potential therapeutic agents (Schlachetzki et al., 2013; Schule et al., 2009; Weykopf et al., 2019). Several models have been described to date, and, although they all come with their strengths and weaknesses (reviewed in Schule et al., 2009; Weykopf et al., 2019), for the purpose of investigating basic disease biology mechanisms and for early drug discovery, a model with physiological/pre-clinical relevance and throughput capabilities is preferred.

LUHMES cells offer such an advantage over other cell models as they are human genome-based lines that are easy to culture, cost efficient and can be differentiated into a homogenous population of DA neurons for the study of DA biology or for the development of assays for PD-associated studies. We have thus exploited such features and developed a novel and physiologically relevant cellular model of PD by overexpressing WT and G2019S LRRK2 in these cells. Despite lacking the relevance of iPSC-derived DA neurons obtained from patients, our model is still a good proxy for modeling PD as LUHMES cells can be easily and quickly differentiated in mature midbrain DA-like neurons, which display DA features. As obtaining and culturing human post-mortem midbrain DA neurons is still challenging, and as the quality and quantity of midbrain DA neurons derived from iPSC differentiation is poor, LUHMES cells offer a valid alternative system to study PD in a physiological setting and to interrogate LRRK2 biology in a relevant context. Finally, our model offers the advantage over iPSC-derived midbrain DA neurons in that it can be easily adapted to HTS as it is simple, homogeneous, cost efficient and scalable, thus permitting the development of robust and reproducible cellular assays for the discovery of new LRRK2-targeted pathways and novel potential therapeutic compounds.

We have shown that expression of mutant LRRK2 causes different disease-relevant phenotypes, such as nuclear, mitochondrial and lysosomal alterations in LUHMES DA-like neurons. The phenotypes that we have identified are consistent with previously published data, which support a LRRK2 role in maintaining nuclear architecture and mitochondria and lysosomal homeostasis, thus further validating the relevance of our cellular model. Alterations in nuclear structure and nuclear membrane function are a common feature of neuronal aging and neuropathologies (Feser and Tyler, 2011; Frost et al., 2016; Iacono et al., 2008; Mertens et al., 2015; Oberdoerffer and Sinclair, 2007; Scaffidi and Misteli, 2006; Worman et al., 2010). Aging, a risk factor for PD (Collier et al., 2011), has been shown to be associated with lamin A/C-dependent nuclear defects (Scaffidi and Misteli, 2006). Accordingly, mimicking aging in PD patient-derived iPSCs by expression of progerin, a truncated lamin A form involved in the Hutchinson-Gilford progeria syndrome, accelerates a multitude of disease-related phenotypes (Miller et al., 2013). With respect to LRRK2, recent studies have reported abnormal nuclear shape and disorganized nuclear envelope structure in neural precursor cells and hippocampal neurons of PD patients carrying the LRRK2 G2019S mutation (Liu et al., 2012; Shani et al., 2019) and in DA neurons of transgenic mice expressing the LRRK2 R1441C mutation (Tsika et al., 2014). While this paper was in preparation, Chen et al. (2020) have shown that LRRK2 G2019S caused an increase in nuclear size in dopaminoceptive striatal spiny projection neurons, and that this phenomenon was regulated by LRRK2 kinase activity. Altogether, these findings have highlighted a key physiological role of LRRK2 in maintaining nuclear morphology and genome integrity and suggest that nuclear abnormalities with LRRK2 mutants are likely due to LRRK2 loss of function. Future understanding of the role of G2019S LRRK2 in underlying abnormal cellular neuronal morphology may provide valuable clues to the pathogenesis and eventual therapy for PD.

It is generally accepted that mitochondrial and lysosomal dysfunction play a role in the etiology and pathophysiology of both familial and sporadic forms of PD. As *LRRK2* is both a familial gene and a susceptibility factor for PD, drugs that could restore LRRK2-mediated mitochondrial defects could be useful in treating familial and idiopathic PD. In addition, as dysfunctional mitochondria and lysosomes have been implicated in the pathology other neurodegenerative diseases (Johri and Beal, 2012; Lezi and Swerdlow, 2012; Lie and Nixon, 2019; Peng et al., 2019), targeting mitochondrial and lysosomal defects may thus open new avenues for the development of therapies for PD, and also for other devastating neurodegenerative disorders. Although we have only investigated three major PD-related phenotypes, as LRRK2 is involved in multiple pathways, our model could be used to interrogate other phenotypes that have been reported in patient-derived cells; for example, abnormal nuclear envelope architecture, altered neurite length and branching, and increased mitochondrial DNA damage.

Numerous preclinical studies reinforce the notion that mitochondrial and lysosomal defects are primary mechanisms of PD (Grunewald et al., 2019; Wallings et al., 2019). However, multiple drug trials targeting mitochondrial impairment have failed to show therapeutic efficacy and this lack of success can be attributed, among other factors, to an incomplete understanding of the molecular pathways driving PD, and the lack of adequate biomarkers to monitor drug efficacy (Lang and Espay, 2018). The precise disease mechanisms involved in mitochondrial and lysosomal dysfunction still need to be fully elucidated, and, in the case of LRRK2, there are many questions that still need to be answered. For example, how do LRRK2 mutants affect mitochondria and lysosome homeostasis? Are these direct or indirect effects? How are these two events linked? In addition, a comprehensive compendium of LRRK2 substrates, interactors and associated signaling pathways is needed. Although a subset of Rab GTPases has been identified as authentic LRRK2 substrates (Dodson et al., 2012; Steger et al., 2016), a continuing search for additional LRRK2 substrates and associated upstream and downstream effectors is of critical importance to understand LRRK2 PD-mediated mechanisms, to identify specific and sensitive disease biomarkers and to develop targeted therapies. Our model system therefore offers the advantage that it can be used to help deciphering the pathways and targets of LRRK2-mediated mitochondrial and lysosomal dysfunction in PD.

It can be argued that LRRK2 overexpression in DA neurons does not model the physiological levels of LRRK2 present in neurons. In addition, it could be argued that LRRK2 overexpression would more likely represent an acute model and would not mirror the progressive, age-dependent molecular pathology of PD. Indeed, our model displays disease-related phenotypes more quickly than PD animal models or patients. However, these features can be advantageous for screening purposes and for the identification of general mechanisms linked to early disease onset and progression. Simplicity is also another limitation of our system, given that many key steps of PD pathogenesis and pathophysiology require the interaction of different cell types (i.e. glia), as well as changes in synaptic and network properties. Consequently, the findings obtained in our model will need to be validated in more complex systems such as co-culture systems and/or 3D cellular models and, eventually, in animal models of PD.

The reduction in LRRK2 levels after ∼6 days of LUHMES neuronal differentiation, which does not allow us to follow the impact of LRRK2 overexpression in fully differentiated LUHMES cells, may be seen as a major drawback of our cellular model. As PD is considered a late-stage PD-associated pathology, the relevance of our early developmental neuronal model to the study of the disease PD could be questionable. However, emerging evidence indicates that PD might also have a neurodevelopment component, and, more importantly, we believe that the developmental period is underscored in PD pathogenesis. Mutations associated with PD are carried throughout life, including especially early brain developmental-critical periods, and may exert significant effects on the establishment and maturation of relevant circuits that impact their function, and perhaps viability, throughout life. As most of the data obtained from cellular and animal models reflect the late and dopamine-depleted states of the disease, where compensatory mechanisms are already in place, we believe that models reflecting the early stage of the disease could be extremely useful to dissect early-phase mechanisms that play a key role in the progression of PD and for understanding which factors contribute to the disease course. Many questions that define the underlying genesis of the neuronal dysregulation and death in disorders like PD remain unanswered, with evidence suggesting a key role for mitochondrial dysfunction. In our study, we showed that mitochondrial phenotypes, which have been described mostly in terminally differentiated neurons, are already detectable in pre-differentiated LUHMES cells. Our findings are in line with those by Walter et al. (2019) and suggest that mitochondrial alterations in LUHMES cells at early stages of differentiation could impact neurogenesis and DA differentiation dynamics in PD. Thus, we believe that our early developmental DA LUHMES PD model can be still relevant to study the early cellular perturbations and cellular adaptations caused by mutant LRRK2 expression.

Our qPCR results suggest that reduction in transgenic *LRRK2* is caused by a decreased basal transcription of *LRRK2* mRNA. In the future, in order to avoid this issue, it would be interesting to explore the effect of using an inducible promoter, different from the TET-off system, to drive conditional *LRRK2* expression or even to consider the use of CRISPR/Cas9 technology to generate mutations in the endogenous gene.

LUHMES cells have been for a long time considered to be hard to transfect, and lentiviral systems have been used for stable GOI integration (Schildknecht et al., 2013). Here, we have described an optimized nucleofection strategy that allows for stable integration of the *LRRK2* genes into the LUHMES pre-neuronal cells. This strategy could be used to introduce other PD genes in the LUHMES background with the goal of creating novel PD models in human DA-like neurons. The discovery of disease-causing genes for PD, such as *LRRK2* and *SNCA*, have facilitated the creation of model systems of the disease by overexpressing disease-driving proteins in a precise cellular context to study their functional consequence. For example, Schildknecht et al. (2013) and Zhang et al. (2014) have created a model of SNCA overexpression in LUHMES cells and have shown the utility of such models for the study of neurotoxicants and the discovery of neuroprotective compounds. The advantage of our AMAXA-based transfection protocol, compared to the one developed by Schildknecht et al. (2013), relies on transfecting the LUHMES cells while proliferating. This allows the generation of stable clones that can subsequently be expanded and banked for use in HTS or different secondary assays.

In conclusion, we believe that our model represents a very useful and manageable human DA-like tool for investigating several aspects of LRRK2 biology and for the discovery of novel therapeutic agents for both familial and sporadic PD, efforts that have been hampered by the lack of high-quality and adequate number of human DA neurons.

## MATERIALS and METHODS

### Plasmids and cell culture

Plasmids used in this work are listed in Table S1. LUHMES cells were obtained from the American Type Culture Collection (catalog no. CRL-2927), tested for mycoplasma contamination, and cultured according to the supplier's recommendations and as previously described (Lotharius et al., 2002), with minor modifications. All experiments in this study were conducted with cells at a passage of 2 to 20. Briefly, cell culture flasks, dishes and plates (six-, 12- and 48-well plates) were manually coated with poly-D-lysine at 2 µg/ml (Sigma-Aldrich, catalog no. P6407) overnight at room temperature, rinsed with sterile water and allowed to fully air dry at room temperature before cell plating. Ninety-six-well plates were bought already pre-coated with poly-D-lysine (Greiner, catalog no. 655946). LUHMES cells were maintained in proliferation medium [Dulbecco's modified Eagle medium/Ham's F-12D medium (advanced DMEM/F-12); GIBCO ThermoFisher Scientific, catalog no. 12634010] containing 1% N2 supplement (GIBCO ThermoFisher Scientific, catalog no .17502048), 1% Glutamax and recombinant human fibroblast growth factor (FGF)-basic (GIBCO ThermoFisher Scientific, catalog no. 13256029) reconstituted at 4 µg/ml in 10 mM Tris-HCl (pH 7.4) plus 0.1% bovine serum albumin (BSA) (Sigma-Aldrich, catalog no. A2058) and added to the proliferation medium immediately before use at a 40 ng/ml final concentration. LUHMES cells were passaged upon reaching no more than 75% confluence. For induction of differentiation, cells were switched to advanced DMEM/F-12 medium plus N2 supplement containing these additional components: 1 µg/ml tetracycline hydrochloride (Sigma-Aldrich, catalog no. T7660), 2 ng/ml recombinant human glial cell-derived neurotrophic factor (GDNF) (R&D Systems, catalog no. 212-GD-010) and 1 mM dbCAMP (Sigma-Aldrich, catalog no. D0627). When indicated in the text, cells were incubated in minimal medium, which was composed of advanced DMEM/F12 supplemented with 1% Glutamax and 1% penicillin and streptomycin.

### *LRRK2* mRNA codon usage assessment

In order to determine whether an mRNA of interest uses rare codons, relative synonymous codon usage (RSCU) values associated with each codon were calculated using GeneDesign 3.0 (Richardson et al., 2010) and human coding sequence (CDS) from RefSeq (NM_ mRNAs–version 20160607; https://www.ncbi.nlm.nih.gov/refseq/). RSCU values are the number of times a particular codon is observed, relative to the number of times that the codon would be observed in the absence of any codon usage bias. In the absence of any codon usage bias, the RSCU value is 1.00. A codon that is used less frequently than expected will have a value of less than 1.00 and vice versa for a codon that is used more frequently than expected. Codon bias of *LRRK2* CDS (GenBank accession number BC117180.1) was defined using GeneDesign with the RSCU values previously calculated. The human *LRRK2* CDS was synthesized and human codon optimized at ThermoFisher Scientific (GeneArt, Regensburg, Germany) and subsequently subcloned into proprietary transposon vectors for transfections.

### Generation of LUHMES cells overexpressing GFP, WT and G2019S LRRK2

Undifferentiated LUHMES cells were trypsinized with trypsin/EDTA 0.025% (GIBCO, catalog no. 25300-054) diluted by half with PBS. Cells were then centrifuged to remove the remaining medium, and 8 million cells were resuspended in 340 µl solution P3 plus supplement of the Nucleofector solution from the AMAXA Basic Nucleofector Kit Primary Mammalian Neurons (Lonza, catalog no. V4XP-3032). Then, 8.5 µg of transposase plasmid was added to this LUHMES master stock. Twenty microliters of the master stock were then aliquoted to each well of the AMAXA nucleofection eight-well strip (Lonza) and 2 µl plasmid solution was added, containing either 0.2 µg eGFP control plasmid, 0.3 µg WT LRRK2 plasmid or 0.3 µg G2019S LRRK2 plasmid. Each condition was run in duplicate. Cells were transfected using the program EM110 of the AMAXA 4D nucleofector system. After nucleofection, 80 µl proliferation medium was added to each well of the strip. Duplicate assays were pooled and were seeded into 12-well plates coated with poly-D-lysine already containing 1.4 ml proliferation medium. Nucleofection efficiency was monitored 2 days later by fluorescence microscopy in GFP-only-expressing cells. Cells were then trypsinized and seeded at 5×10^5^ cells in 35-mm-diameter dishes. A cell pool was obtained by antibiotic selection using 0.2 µg/ml puromycin (InvivoGen, catalog no. ant-pr-1). Single-cell clones were obtained by serially diluting transfected LUHMES in six-well culture plates (Costar, catalog no.3506) coated with poly-D-lysine. Ten, 100 or 1000 cells/well were seeded under 0.2 µg/ml puromycin selection. Isolated clones were identified and picked when the colony began to form a ‘ball’. For picking colonies successfully, trypsin/EDTA diluted at 0.025% in PBS−/− was used to detach the cells. After colony picking, trypsin was quickly inactivated by transferring the colony to minimal medium with 10% bovine serum. After the trypsin-inactivation step, the cells were seeded with proliferation medium in a 48-well culture plate (Costar, catalog no. 3548) coated with poly-D-lysine. Puromycin selection (0.2 µg/ml) was applied after 24 h incubation at 37°C in 5% CO_2_. After proliferation, the clones were transferred to a six-well culture plate coated with poly-D-lysine and further expanded.

### Cell viability assay

Naïve, WT and G2019S LRRK2 LUHMES cell clones L10WT and L14GS were seeded at a density of 10,000 cells/well in 50 µl differentiation medium in 96-well plates and allowed to differentiate for a total of 2 days. Cells were then lysed by the addition of 50 µl Cell TiterGlo reagent (Promega, catalog no. G7573), incubated in the dark for 10 min after a 2-min shaking. Luciferase signal was measured using a PHERAstar microplate reader (BMG Labtech). Each experiment was performed in triplicate (*n*≥8 per cell line). Results from the viability assays are expressed as percentage of the naïve LUHMES cell control±s.d.

### WB

This general procedure was used for all WB experiments, unless specified otherwise. LUHMES were grown and differentiated in poly-D-lysine-coated T75 flasks or six-well plates as follows. First, 8 million cells per T75 flasks or 0.6×10^6^ cells per plate were seeded in medium containing differentiation-inducing factors (see ‘Plasmids and cell culture’ section above). Half of the medium was changed every 2 days. Following differentiation, post-mitotic neurons were harvested for WB. For time-course experiments, LUHMES cells were harvested on the indicated days. Cells were lysed in 30 µl lysis buffer (cell extraction buffer; Invitrogen, catalog no. FNN0011) supplemented with PMSF 1 mM, Phospho stop (Merck, catalog no. 4906837001) and protease inhibitor cocktail (Merck, catalog no. 5892791001) and loaded onto 4-20% Mini-PROTEAN TGX gels (Bio-Rad, catalog no. 4568096) with 1× running buffer (Bio-Rad, catalog no.161-0772). Following transfer, polyvinylidene difluoride (PVDF) membranes (Bio-Rad, catalog no 170-4156) were incubated with the following antibodies in blocking buffer (1× TBS, 0.1% Tween 20 with 5% w/v nonfat dry milk): rabbit anti-LRRK2 monoclonal antibody (Abcam, catalog no. ab133474) at a 1:1000 dilution; mouse anti-LRRK2 antibody (Antibodies Incorporated, Neuromab, catalog no. 75-253, clone N241A/34) at 1:1000 dilution; rabbit anti-LRRK2 phospho S1292 antibody (MJFR-19-7-8) (Abcam, catalog no. ab203181) at 1:500 dilution; rabbit anti-GAPDH antibody (Cell Signaling Technology, catalog no. 2118) at 1:10,000 dilution; rabbit anti-SNCA antibody (Invitrogen, catalog no. 701085) at a 1:1000 dilution; rabbit anti-TH antibody (Merck, catalog no. AB152) at 1:500 dilution; mouse anti-TUJ1 antibody (Biolegend, catalog no. 801202) at a 1:1000 dilution; rabbit anti-RAB10 (Cell Signaling Technology, catalog no. D36C4) at 1:1000 dilution; rabbit anti-p(T73)-RAB10 (Abcam, catalog no. ab230261) at 1:500 dilution; and anti-LAMP2 mouse (Santa Cruz Biotechnology, catalog no. sc18822) at 1:2000 dilution. Secondary antibody detection was performed with donkey anti-rabbit horseradish peroxidase (Jackson ImmunoResearch, catalog no. 711-036-152) at 1:15,000 and goat anti-mouse horseradish peroxidase (Jackson ImmunoResearch, catalog no.115-036-146) at 1:15,000, developed with a chemiluminescent substrate (Super Signal West Femto, ThermoFisher Scientific, catalog no. 34095), and imaged on a ChemiDoc instrument (Bio-Rad). Secondary antibody detection for LAMP2 was performed with rabbit anti-mouse horseradish peroxidase (Jackson ImmunoResearch, catalog no. 315-036-003) at 1:10,000, developed with a chemiluminescent substrate (Super Signal West Dura extended ThermoFisher Scientific, catalog no. 34076), and imaged on a ChemiDoc MP instrument (Bio-Rad). In-gel protein labeling (stain-free technology) was used to normalize protein load, unless specified otherwise. In this case, criterion stain-free gel was activated by exposing the gel to 1 min of UV transillumination with the ChemiDoc MP before imaging. Control proteins were recombinant full-length human his-tagged RAB10 (Novus Biological, catalog no. NBP2-23392) and human full-length LRRK2 (ThermoFisher Scientific, catalog no. 15383806). For detection of total LRRK2 and total RAB10, the Clarity Western ECL substrate (Bio-Rad, catalog no. 1705061) was used. For detection of pRAB10, the Clarity Max Western ECL substrate (Bio-Rad, catalog no. 1705062) was used. For WB in Fig. 2A and Fig. S3, lysates were loaded onto 4-15% Mini-PROTEAN TGX gels (Bio-Rad, catalog no. 456-1086). In addition, for WB in Fig. 2A, before incubation of the membrane with the anti-TH antibody, the membrane was stripped with Restore Blot Stripping Buffer (ThermoFisher Scientific, catalog no. 46430) and, after a blocking step, incubated with mouse anti-TH antibody (Sigma-Aldrich, catalog no. T1299) at a 1:1000 dilution.

The small-molecule LRRK2 kinase inhibitor MLI-2 was synthesized in house. The identity and high purity (i.e. >97.4%) of the compound was confirmed by nuclear-magnetic resonance and mass spectrometry.

### Immunofluorescence

This general protocol was followed unless stated otherwise. LUHMES cells were differentiated in 96-well plates (Greiner, catalog no. 655946) pre-coated with poly-D-lysine. Paraformaldehyde (PFA) (ThermoFisher Scientific, catalog no. 28908) was added to the cells at a concentration of 16% v/v and diluted with cell medium to a final concentration of 4% v/v for 1 h at room temperature. Cells were then permeabilized with 0.1% Triton X-100 in blocking buffer (1% BSA, 5% goat serum in PBS−/−) for 1 h at room temperature. After washing, cells were stained with primary antibodies at 4°C overnight followed by secondary antibodies for 1 h at room temperature. Hoechst 33342 (Invitrogen, USA) was used for nuclear staining. Images were taken using an Operetta CLS high-content imaging system (Perkin Elmer). Primary antibodies used in the study included: mouse monoclonal anti-SNCA (BD Transduction Laboratories, catalog no. 610787) at 1:500 dilution, rabbit monoclonal anti-SNCA 14H2L1 (Invitrogen 701085, batch 242408) at 1:500, mouse anti-MAP2 (Sigma-Aldrich, catalog no. M4403) at 1:500 dilution, mouse anti-TUJ1 (Biolegend, catalog no. 801202) at 1:4000 dilution, mouse anti-LAMP2 (Santa Cruz Biotechnology, catalog no. sc18822) at 1:2000 dilution, mouse anti-LRRK2 clone N241A/34 (Neuromab, catalog no. 75-253, batch 455.7 JD 23) at 1 mg/ml, mouse anti-TOM20 (BD Transduction Laboratories, catalog no. 612278, batch 6210812) at 250 µg/ml. The secondary antibodies used in the study were goat anti-mouse IgG Alexa Fluor 488 (Invitrogen, catalog no. A11001) at 1:200 dilution and goat anti-rabbit Alexa Fluor 555 (Invitrogen, catalog no. A21428, batch 1252795) at 2 mg/ml dilution.

For high-content imaging, WGA AF555 (Molecular Probes, catalog no. W32464) was added before cell permeabilization. For staining of mitochondria, the above procedure was used except for the following modifications: after 1 h incubation with 4% v/v PFA at room temperature, the wells were carefully emptied and 4% PFA in PBS−/− was added for another incubation of 15 min. After several PBS−/− washes, the cells were permeabilized with Triton 0.1% (100% Triton X-100 from Sigma-Aldrich, catalog no. 107K00601) in PBS−/− 20 min at room temperature, washed once with PBS−/− and blocked for 30 min in Odyssey blocking buffer (LICOR, catalog no. 927-40000). The wells were emptied, and mouse anti-TOM20 antibody (BD Transduction Laboratories, catalog no. 612278) diluted at 1:500 in 50/50 Odyssey blocking buffer/PBS Tween 0.1% (Tween 20 stock from Sigma-Aldrich, catalog no. P1379) added for an overnight incubation at 4°C. After several PBS−/− washes, the cells were stained with the goat anti-mouse Alexa Fluor 488 (Invitrogen, catalog no. A11001) secondary antibody for 2 h, and with Hoechst 33342 (Molecular Probes, catalog no. H3570) and Cellmask Orange (Invitrogen, catalog no. C10045) for 20 min. Pictures were taken on an LSM800 confocal microscope (Zeiss). Images were collected with a 40× water objective.

### Quantitative analysis of TH^+^ neurons

Quantification of TH-expressing neurons in naïve, WT and G2019S LRRK2-expressing LUHMES cells at day 1 and day 6 of differentiation was performed using ImageJ. Cells were counted as TH^+^ (green) neurons when TH staining colocalized with nuclear Hoechst staining (blue). All data were presented as the mean±s.d. from two separate experiments and statistically analyzed by one-way analysis of variance (ANOVA) with Turkey's post test for multiple groups. The differences were considered statistically significant when the *P*-value was less than or equal to 0.05 with *n*≥6 from two independent experiments.

### MitoTracker Green mitochondrial staining

LUHMES cells were differentiated in 96-well poly-D-lysine-coated plates (Greiner, catalog no. 655946). Then, the cells were incubated 30 min with 50 nM MitoTracker Green (ThermoFisher Scientific, catalog no. M7514) and Hoechst 33342 (Molecular Probes, H3570). Images were taken on the LSM800 confocal microscope (Zeiss) with a 40× objective and, before imaging, the medium was replaced by HBSS−/− (GIBCO, 14175-053).

### Real-time qPCR

Cells were differentiated in six-well plates for a total of 6 days. At days 0, 1, 2, 3 and 6, cells were collected and RNA was extracted using an RNeasy Plus Mini Kit (Qiagen, USA). The concentration of RNA was measured by a NanoDrop Lite Spetrophotometer (ThermoFisher Scientific). RNA (0.5 µg per sample) was reverse transcribed to cDNA using a High-Capacity cDNA Reverse Transcription Kit with RNase Inhibitor (ThermoFisher Scientific). Real-time PCR was run in a StepOnePlus Real-Time PCR System (Applied Biosystems, USA). Reverse transcriptase reaction mixtures were prepared as per the manufacturer's protocol, and reactions were performed by incubating 0.5 µg RNA at 25°C for 10 min then 120 min at 37°C, followed by inactivation at 85°C for 5 min. Primers sequences used in the study are Custom plus Taqman RNA assay (SM) LRRK2 codon optimized sequence primer catalog number 4441114 and GAPDH Taqman gene expression assay hs99999905-m1 (ThermoFisher Scientific).

### Quantitative imaging of nuclear, mitochondrial and lysosomal morphology

For quantitative imaging of nuclear morphology, LUHMES cells were seeded at 3500 cells/well in 96-well plates. After 3 days of differentiation, Nuclight Rapid Red Reagent for live cell nuclear labeling (Sartorius, catalog no. 4717) was added at 1:2000 final dilution in medium following the supplier instructions. Images were collected using an Incucyte S3 Live Cell Imaging System (Essen Bioscience) 1 h after dye loading using one-phase contrast and one fluorescent channel (excitation/emission wavelength of 655/681 nm) and a 10× objective. Red eccentricity object average was measured with the Incucyte analysis software.

For quantitative imaging of mitochondrial morphology, LUHMES cells were cultured in 80 cm^2^ flasks (ThermoFisher Scientific, catalog no.178905) coated with poly-D-lysine until 70% confluence was reached. Cells were then detached by addition of trypsin/EDTA in PBS−/− (GIBCO, catalog no. 25300-054), which was diluted 1:1 with PBS for a 0.025% concentration and incubated for 5 min at 37°C. Cells were collected in Minimal Medium, Advanced DMEM/F12 medium (GIBCO Invitrogen), counted and centrifuged at 200 ***g*** for 5 min at room temperature. The cell pellet was resuspended in differentiation medium, and cells were seeded at a density of 20,000 cells/well in pre-coated 96-well plates (Greiner, catalog no. 655946). Medium was changed after 2 days. For these experiments, differentiation was continued for a total of 3 days. Cells were fixed by the addition of 16% PFA (ThermoFisher Scientific, catalog no. 28908) to the plate wells, to end with 4% PFA in the medium. After 1 h at room temperature, the wells were carefully emptied, and 4% PFA in PBS−/− was added for another incubation of 15 min. After several PBS−/− washes, the cells were permeabilized with Triton 0.1% (100% Triton X-100 from Sigma-Aldrich, catalog no. 107K00601) in PBS−/− for 20 min at room temperature, washed once with PBS−/− and blocked for 30 min in Odyssey blocking buffer (LICOR, catalog no. 927-40000). The wells were emptied, and mouse anti-TOM 20 antibody (BD, catalog no. 612278) diluted at 1:500 in 50/50 Odyssey blocking buffer/PBS Tween 0.1% (Tween 20 stock from Sigma-Aldrich, catalog no. P1379) was added for an overnight incubation at 4°C. After several PBS−/− washes, the cells were stained with the secondary antibody goat anti-mouse Alexa Fluor 488 (Invitrogen, catalog no. A11001) for 2 h, 20 min with Hoechst 33342 (Molecular Probes, catalog no. H3570) and Cellmask Orange (Invitrogen, catalog no. C10045). Pictures were taken on the Operetta CLS high-content imaging system (Perkin Elmer) using a 40× water objective. The algorithm to analyze mitochondria morphological changes, was developed with the Harmony 4.9 analysis software (Perkin Elmer).

For quantitative imaging of lysosomal morphology, LUHMES cells were differentiated in 96-well plates (Greiner, catalog no. 655946) pre-coated with poly-D-lysine and fixed, permeabilized and stained as indicated in the ‘Immunofluorescence’ section above. An anti-LAMP2 mouse (Santa Cruz Biotechnology, catalog no. sc18822) primary monoclonal antibody was used at 1:2000 dilution. Secondary antibody was goat anti-mouse IgG Alexa Fluor 488 (Invitrogen) at 1:200 dilution. Images were taken using an Operetta CLS high-content imaging system (Perkin Elmer) using a 40× water objective. At least 45 fields per well on a 96-well plate were imaged per condition. Each condition was run in triplicate wells/plate. Detection of the nuclei was done by Hoechst 33342 staining. Spots were retained for quantitative analysis if their area was higher than 15 px^2^ and a corrected intensity higher than 300.

### Statistical analysis

For all experiments, values were expressed as the mean±s.d. Data were analyzed by one-way ANOVA with factor cell type followed by Tukey's adjustment for multiple groups in order to compare clones with naïve cells and to compare both clones. Heterogeneity of variances was managed with group=cell type option. The relative significance for each of the reported differences is specified as *P*-values and is listed in the figure legends and represented graphically within the figures. *P*<0.05 was considered statistically significant.

## Supplementary Material

Supplementary information
