# Supplementary material for: Development of a physiologically relevant and easily scalable LUHMES cell-based model of G2019S LRRK2-driven Parkinson's disease
Source: Dis Model Mech. 2021 Jun 11;14(6):dmm048017. doi: 10.1242/dmm.048017 (PMC8214734; doi:10.1242/dmm.048017)
Supplement: Supplementary information [file dmm-14-048017-s1.pdf]

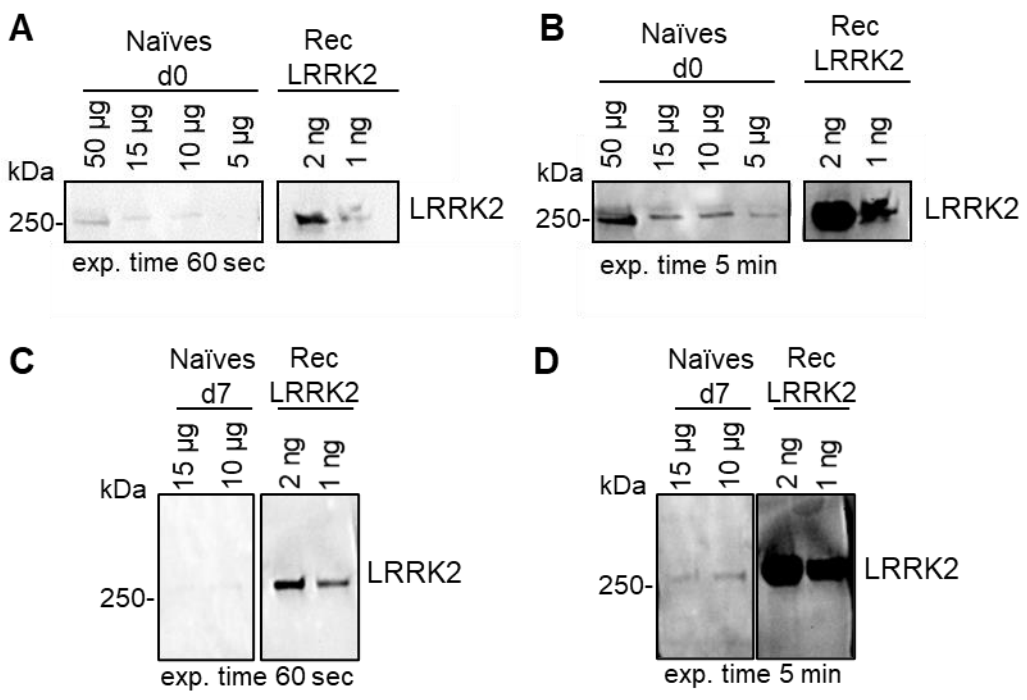

Fig. S1. Naïve LUHMES cells express endogenous LRRK2. (**A**) Proliferating (d0) naïve LUHMES cells were lysed and different amounts of total protein lysate (5, 10, 15 and 50 µg) were loaded on the gel. Endogenous LRRK2 protein is visible by WB analysis of naïve LUHMES cell lysates when the total protein loaded is about 50 µg, if a short exposure time is used (e.g. 60 seconds). Recombinant LRRK2 protein (Rec LRRK2) was used as antibody control. Results are representative of two independent experiments. (**B**) Conditions as in (**A**), but exposure time increased to 5 minutes. Endogenous LRRK2 protein is visible by WB of naïve LUHMES cell lysates when the total protein loaded is higher than 5 µg, if a long exposure times is used (e.g. 5 minutes). Recombinant LRRK2 protein (Rec LRRK2) was used as antibody control. Results are representative of two independent experiments. (**C**-**D**) Differentiated (d7) naïve LUHMES cells were lysed and different amounts of total protein lysate (10 and 15 µg) were loaded on the gel. Endogenous LRRK2 expression is not visible at short exposure time, but detectable at long exposure times when the total protein lysate is ≤15 µg. Recombinant LRRK2 protein (Rec LRRK2) was used as antibody control. Results are representative of two independent experiments. The protein corresponding MW (in kDa) are indicated on the left sides of the images.

Disease Models & Mechanisms • Supplementary information

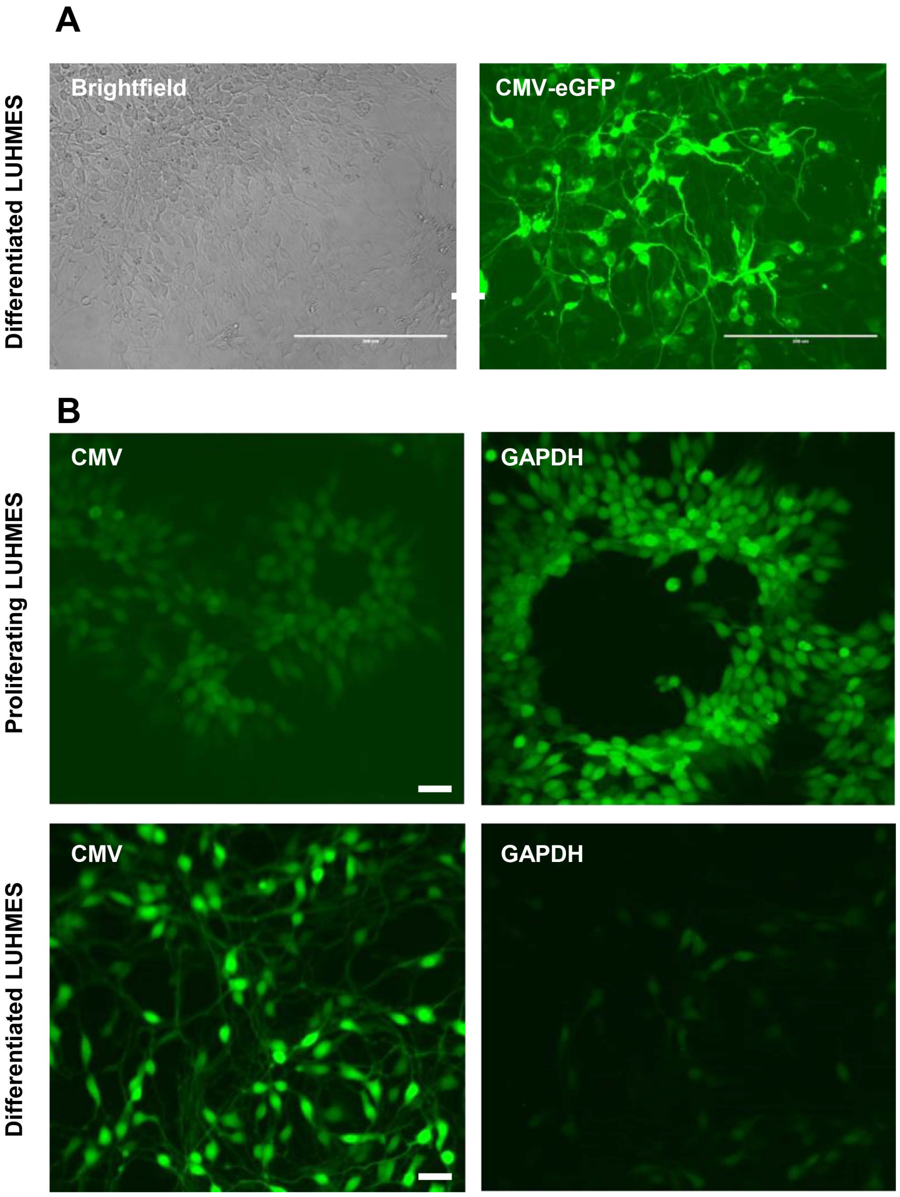

Fig. S2. Optimization of AMAXA nucleofection protocol in proliferating LUHMES cells using GFP-expressing plasmids. **(A)** Brightfield (left) and fluorescence (right) images of LUHMES cells nucleofected with a plasmid encoding for eGFP under the control of the *CMV* promoter before antibiotic selection. **(B)** Representative images of proliferating (upper panels) and differentiated (day 6; lower panels) naïve LUHMES cells nucleofected with plasmids encoding for eGFP under the control of the *CMV* and *GAPDH* promoters. Cells were selected with puromycin and then fixed and imaged. eGFP expression was stronger in proliferating cells when the *GAPDH* promoter was used, whereas the *CMV* promoter led to a higher eGFP expression in differentiated cells.

Disease Models & Mechanisms • Supplementary information

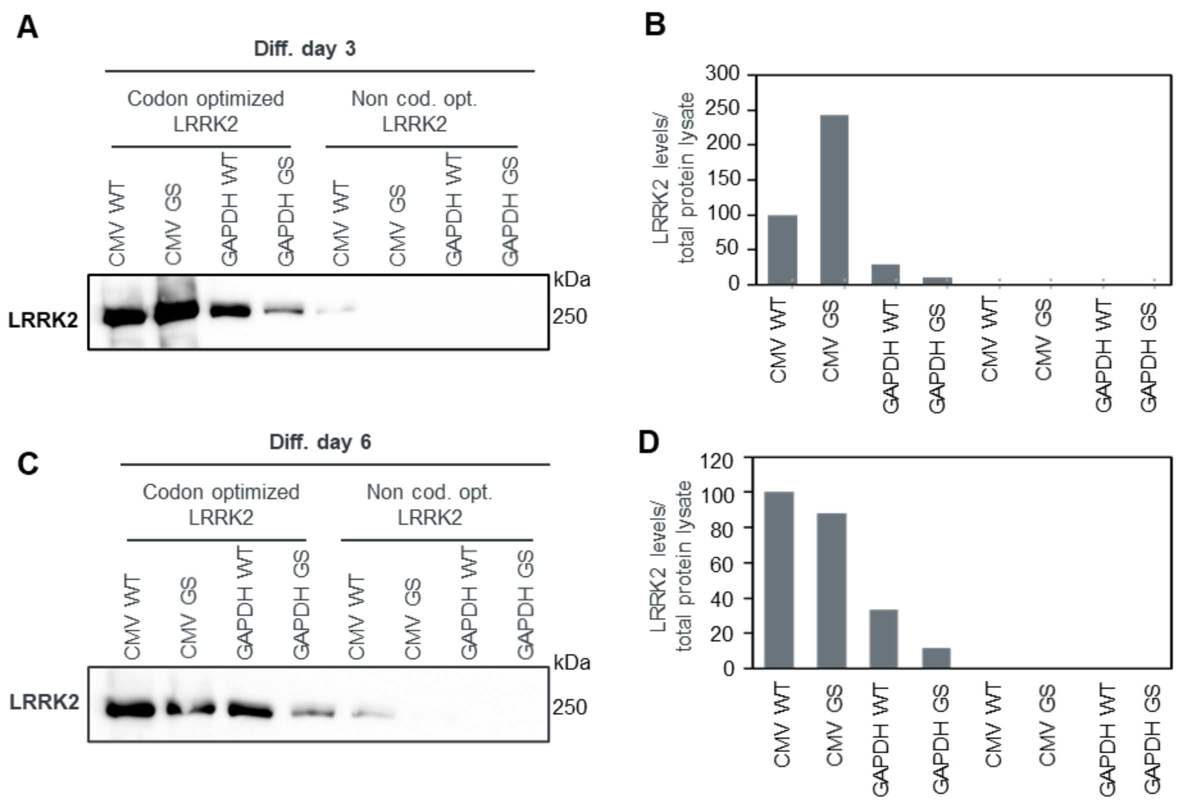

Fig. S3. Generation of LUHMES cells expressing codon-optimized WT and G2019S LRRK2. (**A** and **C**) WB analysis of LUHMES cells expressing codon optimized and non-codon optimized (non-cod. opt.) WT and G2019S (GS) LRRK2 under the control of the CMV and GAPDH promoters at days 3 (**A**) and 6 (**C**) of differentiation. Codon optimization of *LRRK2* DNA ensued robust LRRK2 protein expression. Results are representative of two independent experiments. The protein corresponding MW (in kDa) are indicated on the right side of the figures. (**B** and **D**) Densiometric analysis of LRRK2 levels normalized to total protein samples.

Disease Models & Mechanisms • Supplementary information

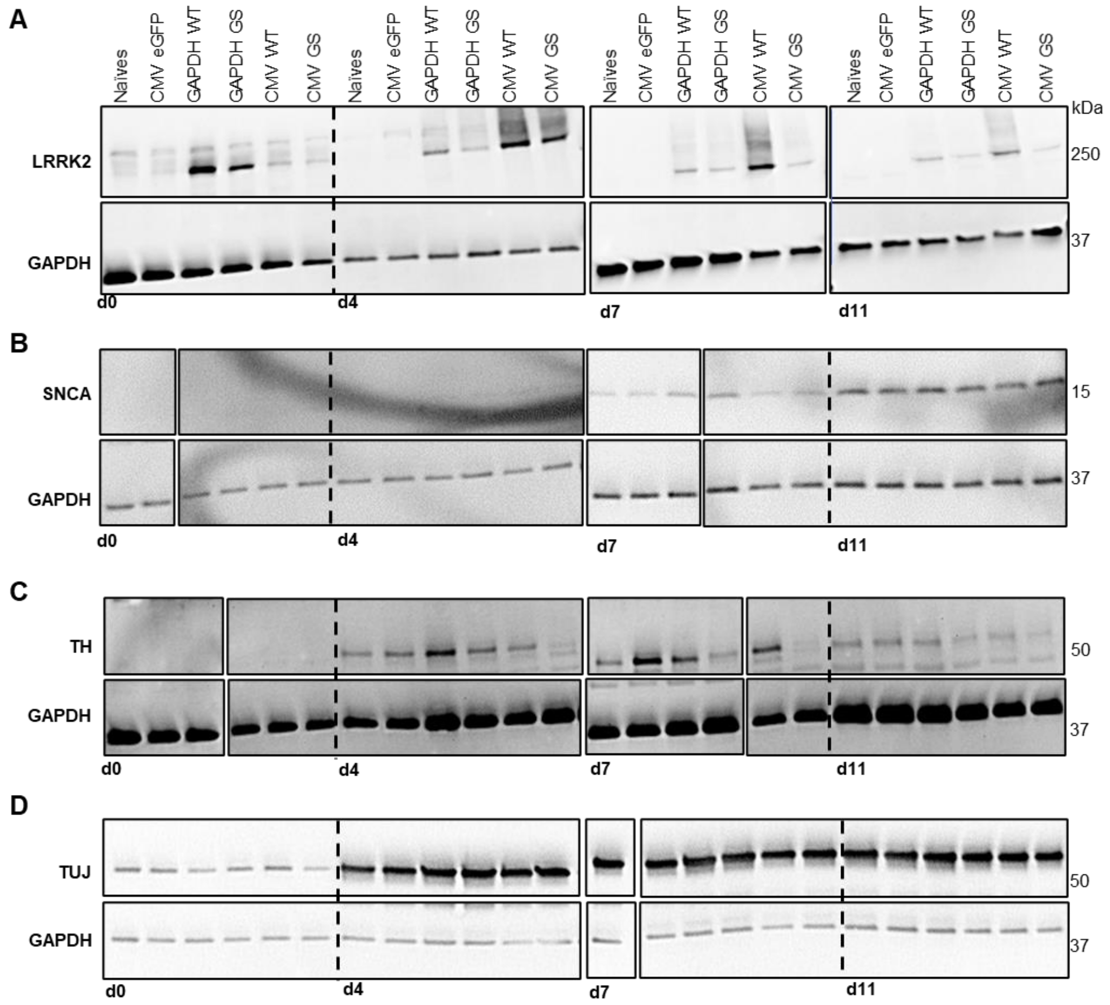

Fig. S4. Kinetics of LRRK2 and neuronal marker expression in differentiating LUHMES cells. (**A - D**) WB analysis of LRRK2 and different neuronal markers during an 11-day LUHMES cell differentiation period. The housekeeping gene GAPDH is used as loading control. The protein corresponding MW (in kDa) are indicated on the right side of the figure. (**A**) LRRK2 levels become more important during differentiation, if LRRK2 expression is under the *CMV* promoter. Also, LRRK2 levels decrease during LUHMES differentiation, with the mutant being more strongly affected. (**B**) Analysis of α-synuclein (SNCA) expression during LUHMES cell differentiation. SNCA levels are barely visible at differentiation day 4 at the exposure time chosen and increase to reach a plateau by day 7. (**C**) The dopaminergic marker tyrosine hydroxylase (TH) is present early during LUHMES cell differentiation (already at day 4) and remain constant during the entire differentiation period (day 11). Expression of this marker is reduced in the G2019S LRRK2 cells. (D) βIII-tubulin (TUJ) is visible already in undifferentiated (day 0) LUHMES cells and its expression becomes stronger during differentiation, reaching a plateau by day 4.

Disease Models & Mechanisms • Supplementary information

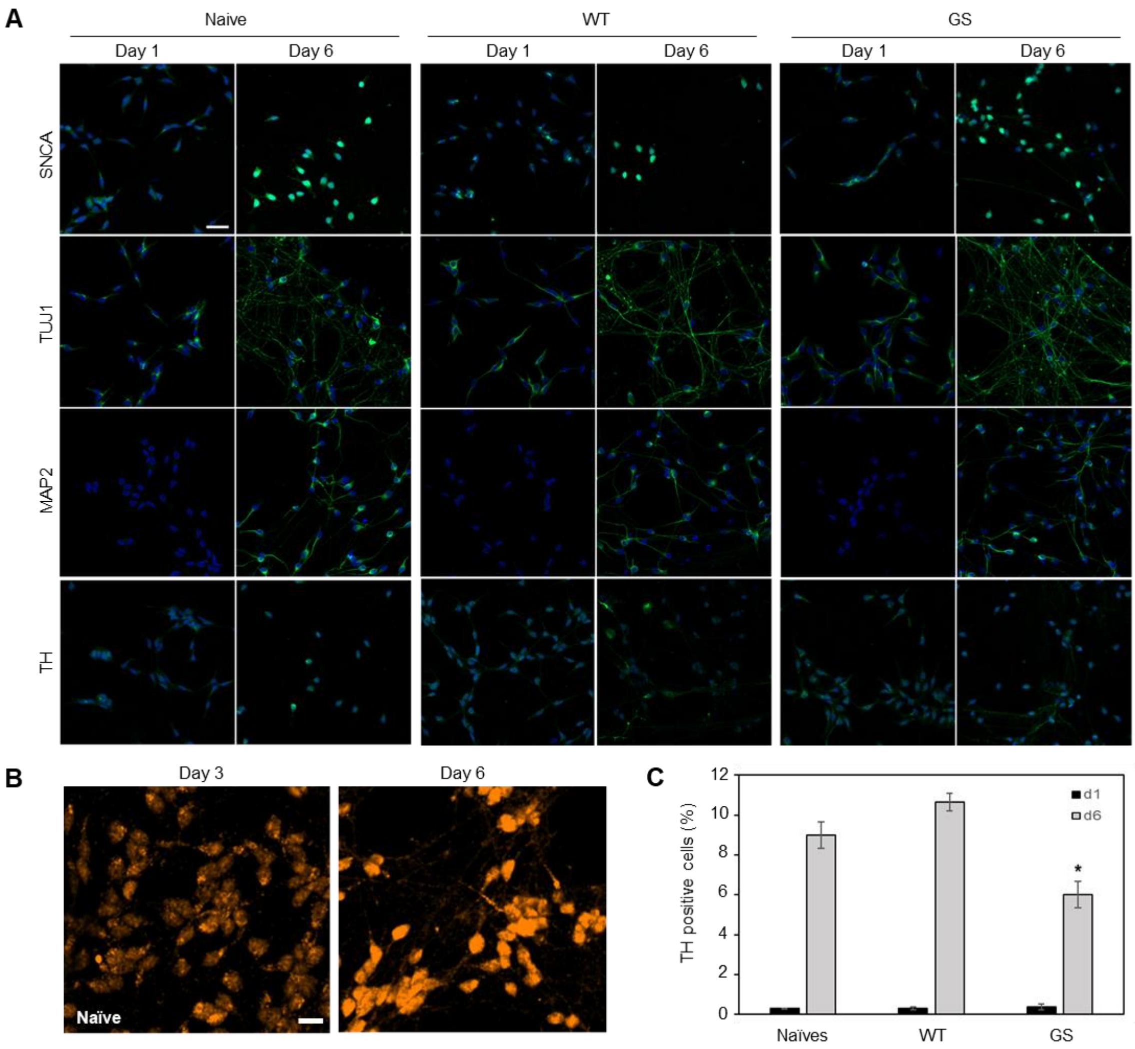

Fig. S5. Immunofluorescence analysis of neuronal markers in early and fully differentiated LUHMES cells. (**A**) Naïve LUHMES cells and LUHMES cell pools over-expressing codon-optimized WT and G2019S (GS) LRRK2 under the control of the *CMV* promoter, were differentiated for one (d1) or 6 days (d6). Cells were then fixed and stained with the neuronal markers α-synuclein (SNCA), β-III tubulin (TUJ1) microtubule-associated protein 2 (MAP2) and tyrosine hydroxylase (TH). Expression of SNCA, MAP2 and TH was visible in differentiated cells, whereas TUJ1 was present already in undifferentiated cells and its levels increased during differentiation. SNCA is mainly seen in the cell bodies at the exposure time chosen. A mouse monoclonal anti-α-synuclein (BD Transduction Laboratories, clone 42) was used. Hoechst 33342: blue; SNCA, TUJ1, MAP2 and TH: green. Scale bar represents 50 µm. (**B**) Representative images of differentiated [day 3 (d3) and day 6 (d6)] naive LUHMES cells. Cells were fixed, permeabilized, and stained with a rabbit monoclonal anti-α-synuclein antibody (Invitrogen, clone 14H2L1). For direct comparison, both images were taken with the same exposure time. α-synuclein levels increased with differentiation times and α-synuclein was visible in the neurites at the late differentiation time. α-synuclein, orange. Scale

Disease Models & Mechanisms • Supplementary information

bar represents 20 µm. (**C**) Quantification of TH+ cells among the total number of Hoechst-stained cells from 6 to 8 images. GS = G2019S LRRK2. All data expressed as mean ± SD from two separate experiments. Raw data were first analyzed using one-way ANOVA and then Tukey's post-test was performed to compare all treatment groups. Differences with $^{*}P \leq 0.05$ were considered significantly different with $n \geq 6$ from two independent experiments.

,

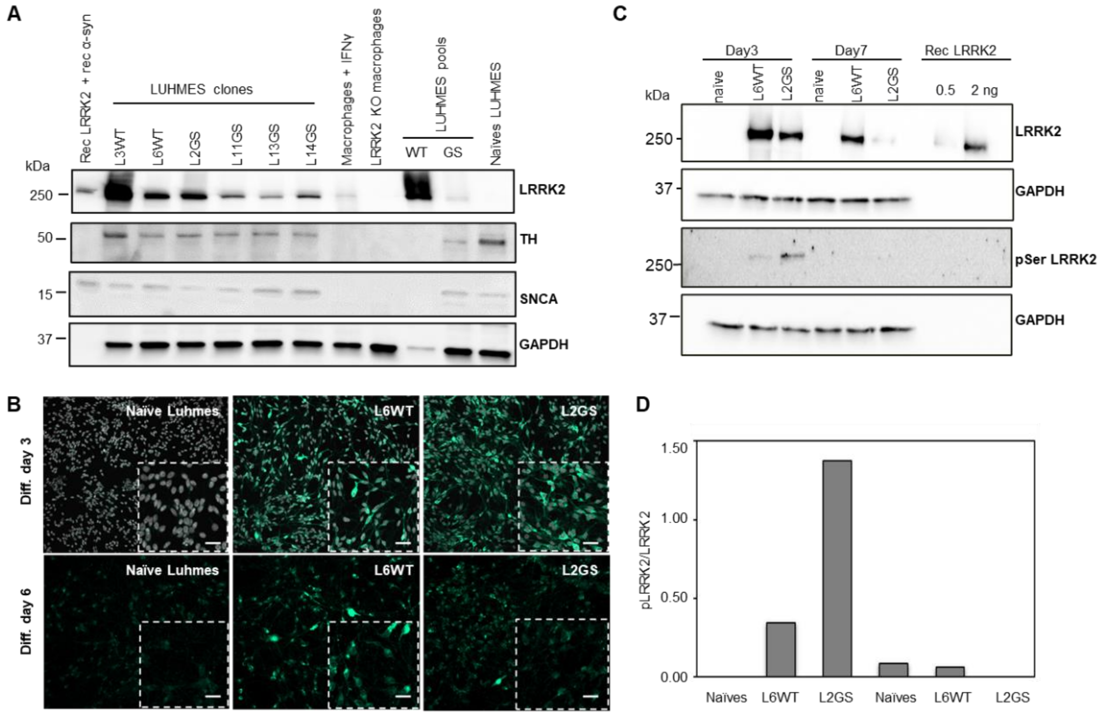

Fig. S6. Western Blot and immunofluorescence characterization of LUHMES cell clones expressing codon-optimized WT and G2019S LRRK2. (**A**) The levels of LRRK2 protein together with the dopaminergic markers tyrosine hydroxylase (TH) and α-synuclein (SNCA) were analyzed by WB in two WT (L3WT and L6WT) and four G2019S (L2GS, L11GS, L13GS, and L14GS) clones differentiated for 3 days. LRRK2 was clearly visible in all clones. L6WT and L2GS clones expressed similar LRRK2 levels at this differentiation time. LRRK2 expression in these clones was under the *CMV* promoter. All clones were able to normally differentiate in dopaminergic-like neurons as inferred by the expression of dopaminergic markers TH and SNCA. To validate the LRRK2 antibody, recombinant LRRK2 protein (Rec LRRK2), WT and naïve LUHMES pool lysates and macrophages treated with IFNγ were used as LRRK2 positive controls, whereas LRRK2 KO macrophages were used as LRRK2 negative control. Recombinant α-synuclein (rec α-syn) protein was used to validate the α-synuclein antibody. The housekeeping protein GAPDH was used as loading control. The position and size (in kDa) of molecular weight standards are indicated on the left side of the panel. The displayed experiment is representative of two independent experiments. Note that for the sample named “LUHMES pools – WT” TH and SNCA levels are not detected as we loaded a much lower total protein lysate for this sample (see GAPDH levels) in order to avoid a big smear from the LRRK2 band. (**B**) WT and G2019S LRRK2 expression was confirmed in LUHMES clones by IF analysis. Naïve, WT (L6WT) and G2019S (L2GS) LUHMES clones were differentiated for three and six days and then fixed, permeabilized and stained with the anti-LRRK2 NeuroMab clone N241A/34. Analogously to the WB results, LRRK2 was not detected

Disease Models & Mechanisms • Supplementary information

in naïve LUHMES cells, but was strongly and homogeneously expressed in the WT and G2019S clones. LRRK2 is shown in green; nuclei are shown without pseudocolor. For direct comparison, images were taken with the same exposure time. Scale bars represent 25 µm. Results representative of three independent experiments. (**C**) LRRK2 expression decreases with the differentiation of LUHMES. LUHMES cells were differentiated for 3 and 7 days and then collected for WB analysis of LRRK2 and Ser1292 phosphorylation. A robust expression of both WT and G2019S LRRK2 was visible at day 3 of differentiation, but LRRK2 levels were reduced at day 7 of differentiation, with the G2019S being more affected. LRRK2 Ser1292 phosphorylation was detected at day 3 of differentiation and was more pronounced for the mutant. Recombinant LRRK2 (rec LRRK2) was used as control. GAPDH was used to ensure equal loading. The position and size (in kDa) of molecular weight standards are indicated on the left side of the panel. The displayed experiment is representative of three independent experiments. (**D**) Densiometric analysis of pSer1292 LRRK2 shown in (**C**) normalized to total LRRK2 protein.

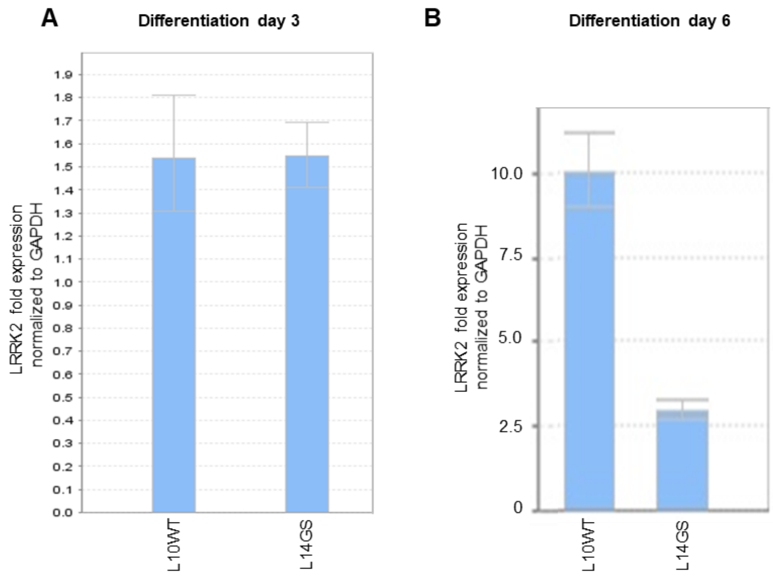

Fig. S7. Levels of *LRRK2* mRNA in differentiated LUHMES clones, as determined by RT-qPCR. (A) mRNA was extracted to quantify *LRRK2* and GAPDH mRNA using quantitative RT-PCR (qRT-PCR). *LRRK2* mRNA levels were measured in differentiated (day 3) LUHMES clones L10WT and L14GS and were found to be equivalent at this time point. *LRRK2* fold mRNA expression was normalized to *GAPDH* levels. The displayed experiment is representative of three independent experiments. Bars show mean ± SD. **(B)** mRNA levels of G2019S *LRRK2* decreased at day 6 of differentiation. *LRRK2* fold mRNA expression was normalized to *GAPDH* levels. The displayed experiment is representative of three independent experiments. Bars show mean ± SD.

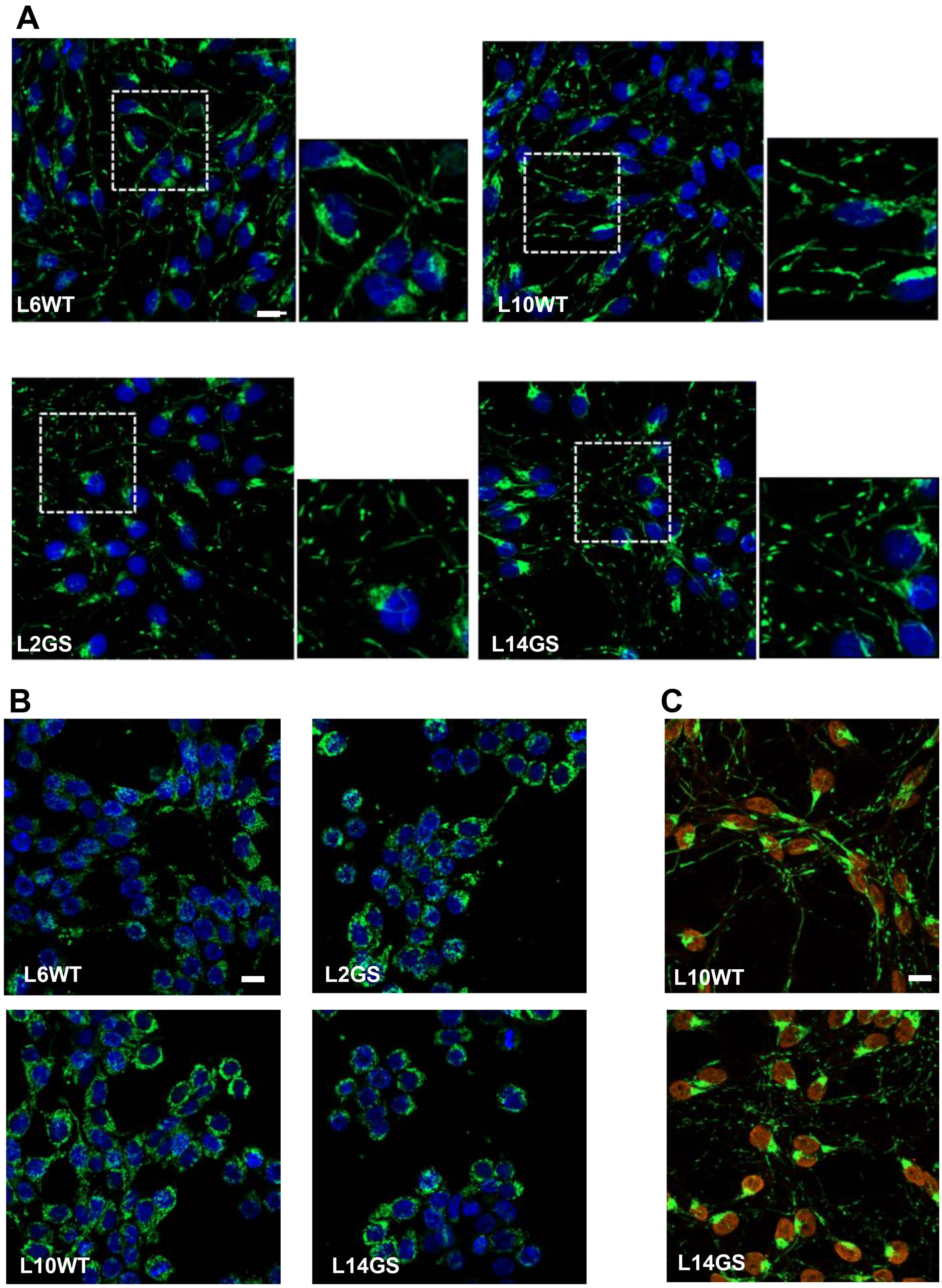
A
L6WT
L10WT
L2GS
L14GS
B
L6WT
L2GS
L10WT
L14GS
C
L10WT
L14GS

Fig. S8. LRRK2 G2019S causes mitochondrial fragmentation in LUHMES cells. **(A)** MitoTracker Green staining of two WT (L6WT and L10WT) and two G2019S (L2GS and L14GS) clones at day 3 of differentiation confirms the data obtained with TOM20 staining: G2019S-expressing clones present a higher mitochondrial fragmentation than WT clones. MitoTracker, green; Hoechst 33342: blue. Scale bar represents 10 µm. The displayed experiment is representative of three independent experiments. **(B)** MitoTracker Green staining of two couples of undifferentiated WT and G2019S clones, as indicated in panel A, show that mitochondria are clumped around the nuclei and no clear difference in mitochondrial morphology is detected. MitoTracker, green; Hoechst 33342: blue. Scale bar represents 10 µm. The displayed experiment is representative of three independent experiments. **(C)** WGA 11 and TOM20 staining of day 3 differentiated WT and G2019S clones. MitoTracker, green; WGA: orange. Scale bar represents 10 µm. The displayed experiment is representative of three independent experiments.

Table S1. List of plasmids used to transfect LUHMES cells.

Construct #	Plasmid name	Resistance	Stock conc (µg/µl)	µg/assay
**1**	CMV cod opt WT	Puromycin	0,173	0,3114
**2**	CMV cod opt GS	Puromycin	0,172	0,3096
**3**	GAPDH cod opt WT	Puromycin	0,148	0,2664
**4**	GAPDH cod opt GS	Puromycin	0,176	0,3168
**5**	CMV eGFP	Puromycin	0,108	0,1944
**6**	GAPDH eGFP	Puromycin	0,124	0,2232
**7**	Transposase	NA	2,27	0.5

cod opt = codon optimized; NA = not applicable

*§cod opt, codon optimized; *NA, not applicable*

Table S2. WT vs G2019S codon optimized LRRK2 DNA sequences.

Score	Expect	Identities	Gaps	Strand
13995 bits(7578)	0.0	7582/7584(99%)	0/7584(0%)	Plus/Plus

```
Query  1    ATGGCCAGCGGCTCCTGCCAGGGCTGCGAGGAAGATGAGGAAACCCTGAAGAAACTGATC  60
Sbjct  1    ATGGCCAGCGGCTCCTGCCAGGGCTGCGAGGAAGATGAGGAAACCCTGAAGAAACTGATC  60

Query  61   GTGCGGCTGAACAACGTGCAGGAAGGCAAGCAGATCGAGACACTGGTGCAGATCCTGGAA  120
Sbjct  61   GTGCGGCTGAACAACGTGCAGGAAGGCAAGCAGATCGAGACACTGGTGCAGATCCTGGAA  120

Query  121  GATCTGCTGGTGTTCACCTACAGCGAGCACGCCAGCAAGCTGTTCCAGGGCAAGAACATC  180
Sbjct  121  GATCTGCTGGTGTTCACCTACAGCGAGCACGCCAGCAAGCTGTTCCAGGGCAAGAACATC  180

Query  181  CACGTGCCCCTGCTGATTGTGCTGGACAGCTACATGCGGGTGGCCAGCGTGCAGCAAGTG  240
Sbjct  181  CACGTGCCCCTGCTGATTGTGCTGGACAGCTACATGCGGGTGGCCAGCGTGCAGCAAGTG  240

Query  241  GGATGGTCCCTGCTGTGCAAGCTGATCGAAGTGTGCCCCGGCACCATGCAGAGCCTGATG  300
Sbjct  241  GGATGGTCCCTGCTGTGCAAGCTGATCGAAGTGTGCCCCGGCACCATGCAGAGCCTGATG  300

Query  301  GGACCTCAGGACGTGGGCAACGACTGGGAGGTGCTGGGAGTGCACCAGCTGATCCTGAAG  360
Sbjct  301  GGACCTCAGGACGTGGGCAACGACTGGGAGGTGCTGGGAGTGCACCAGCTGATCCTGAAG  360

Query  361  ATGCTGACCGTGCACAACGCCAGCGTGAACCTGAGCGTGATCGGCCTGAAAACCCTGGAC  420
Sbjct  361  ATGCTGACCGTGCACAACGCCAGCGTGAACCTGAGCGTGATCGGCCTGAAAACCCTGGAC  420

Query  421  CTGCTGCTGACCAGCGGCAAGATCACACTGCTGATTCTGGACGAGGAATCCGACATCTTC  480
Sbjct  421  CTGCTGCTGACCAGCGGCAAGATCACACTGCTGATTCTGGACGAGGAATCCGACATCTTC  480

Query  481  ATGCTGATCTTCGACGCCATGCACAGCTTCCCCGCCAACGACGAGGTGCAGAAGCTGGGC  540
Sbjct  481  ATGCTGATCTTCGACGCCATGCACAGCTTCCCCGCCAACGACGAGGTGCAGAAGCTGGGC  540

Query  541  TGTAAAGCCCTGCACGTGCTGTTCGAGCGGGTGTCCGAGGAACAGCTGACCGAGTTCGTG  600
Sbjct  541  TGTAAAGCCCTGCACGTGCTGTTCGAGCGGGTGTCCGAGGAACAGCTGACCGAGTTCGTG  600

Query  601  GAAAACAAGGACTACATGATCCTGCTGAGCGCCCTGACCAACTTCAAGGATGAGGAAGAG  660
Sbjct  601  GAAAACAAGGACTACATGATCCTGCTGAGCGCCCTGACCAACTTCAAGGATGAGGAAGAG  660

Query  661  ATCGTGCTGCATGTGCTGCACTGCCTGCACAGCCTGGCCATCCCTTGCAACAACGTGGAA  720
Sbjct  661  ATCGTGCTGCATGTGCTGCACTGCCTGCACAGCCTGGCCATCCCTTGCAACAACGTGGAA  720

Query  721  GTGCTGATGAGCGGCAACGTGCGGTGCTACAACATCGTGGTGGAAGCCATGAAGGCCTTT  780
```

Disease Models & Mechanisms • Supplementary information

Sbjct 721 GTGCTGATGAGCGGCAACGTGCGGTGCTACAACATCGTGGTGGAAGCCATGAAGGCCTTT 780

Query 781 CCCATGAGCGAGCGGATCCAGGAAGTGTCCTGCTGCCTGCTGCATCGGCTGACCCTGGGC 840
Sbjct 781 CCCATGAGCGAGCGGATCCAGGAAGTGTCCTGCTGCCTGCTGCATCGGCTGACCCTGGGC 840

Query 841 AACTTCTTCAACATCCTGGTGCTGAATGAGGTGCACGAGTTTGTCGTGAAGGCCGTGCAG 900
Sbjct 841 AACTTCTTCAACATCCTGGTGCTGAATGAGGTGCACGAGTTTGTCGTGAAGGCCGTGCAG 900

Query 901 CAGTACCCCGAGAATGCCGCCCTGCAGATCAGCGCCCTGTCTTGTCTGGCCCTGCTGACC 960
Sbjct 901 CAGTACCCCGAGAATGCCGCCCTGCAGATCAGCGCCCTGTCTTGTCTGGCCCTGCTGACC 960

Query 961 GAAACCATATTCCTGAACCAGGACCTGGAAGAGAAGAACGAGAACCAGGAAAACGACGAC 1020
Sbjct 961 GAAACCATATTCCTGAACCAGGACCTGGAAGAGAAGAACGAGAACCAGGAAAACGACGAC 1020

Query 1021 GAGGGCGAAGAGGATAAGCTGTTCTGGCTGGAAGCCTGCTACAAGGCCCTGACCTGGCAC 1080
Sbjct 1021 GAGGGCGAAGAGGATAAGCTGTTCTGGCTGGAAGCCTGCTACAAGGCCCTGACCTGGCAC 1080

Query 1081 CGGAAGAACAAACATGTGCAGGAAGCCGCCTGCTGGGCCCTGAACAATCTGCTGATGTAC 1140
Sbjct 1081 CGGAAGAACAAACATGTGCAGGAAGCCGCCTGCTGGGCCCTGAACAATCTGCTGATGTAC 1140

Query 1141 CAGAACTCCCTGCACGAGAAGATCGGCGACGAGGACGGCCACTTTCCTGCCCACCGGGAA 1200
Sbjct 1141 CAGAACTCCCTGCACGAGAAGATCGGCGACGAGGACGGCCACTTTCCTGCCCACCGGGAA 1200

Query 1201 GTGATGCTGAGCATGCTGATGCACTCCAGCTCCAAAGAGGTGTTCCAGGCCAGCGCCAAC 1260
Sbjct 1201 GTGATGCTGAGCATGCTGATGCACTCCAGCTCCAAAGAGGTGTTCCAGGCCAGCGCCAAC 1260

Query 1261 GCCCTGAGTACCCTGCTGGAACAGAACGTGAACTTTCGGAAAATCCTGCTGTCCAAGGGC 1320
Sbjct 1261 GCCCTGAGTACCCTGCTGGAACAGAACGTGAACTTTCGGAAAATCCTGCTGTCCAAGGGC 1320

Query 1321 ATCCACCTGAACGTGCTGGAACTGATGCAGAAGCACATCCACAGCCCCGAGGTGGCCGAG 1380
Sbjct 1321 ATCCACCTGAACGTGCTGGAACTGATGCAGAAGCACATCCACAGCCCCGAGGTGGCCGAG 1380

Query 1381 AGCGGATGCAAAATGCTGAACCACCTGTTCGAGGGCAGCAACACCAGCCTGGACATCATG 1440
Sbjct 1381 AGCGGATGCAAAATGCTGAACCACCTGTTCGAGGGCAGCAACACCAGCCTGGACATCATG 1440

Query 1441 GCCGCCGTGGTGCCCAAGATCCTGACAGTGATGAAGCGGCACGAGACAAGCCTGCCCGTG 1500
Sbjct 1441 GCCGCCGTGGTGCCCAAGATCCTGACAGTGATGAAGCGGCACGAGACAAGCCTGCCCGTG 1500

Query 1501 CAGCTGGAAGCTCTGAGAGCCATCCTGCACTTCATCGTGCCCGGCATGCCCGAGGAAAGC 1560
Sbjct 1501 CAGCTGGAAGCTCTGAGAGCCATCCTGCACTTCATCGTGCCCGGCATGCCCGAGGAAAGC 1560

Query 1561 AGAGAGGACACCGAGTTCCACCACAAGCTGAACATGGTCAAGAAGCAGTGCTTCAAGAAC 1620

Disease Models & Mechanisms • Supplementary information

Sbjct 1561 AGAGAGGACACCGAGTTCCACCACAAGCTGAACATGGTCAAGAAGCAGTGCTTCAAGAAC 1620

Query 1621 GACATTCACAAGCTGGTGCTGGCCGCCCTGAATCGGTTCATCGGCAACCCCGGCATCCAG 1680
Sbjct 1621 GACATTCACAAGCTGGTGCTGGCCGCCCTGAATCGGTTCATCGGCAACCCCGGCATCCAG 1680

Query 1681 AAATGTGGCCTGAAAGTGATCAGCAGCATCGTGCACTTCCCCGACGCCCTGGAAATGCTG 1740
Sbjct 1681 AAATGTGGCCTGAAAGTGATCAGCAGCATCGTGCACTTCCCCGACGCCCTGGAAATGCTG 1740

Query 1741 TCTCTGGAAGGCGCCATGGACAGCGTGCTGCACACACTGCAGATGTACCCCGACGATCAG 1800
Sbjct 1741 TCTCTGGAAGGCGCCATGGACAGCGTGCTGCACACACTGCAGATGTACCCCGACGATCAG 1800

Query 1801 GAAATCCAGTGCCTGGGCCTGTCCCTGATCGGCTACCTGATCACCAAGAAAAACGTGTTC 1860
Sbjct 1801 GAAATCCAGTGCCTGGGCCTGTCCCTGATCGGCTACCTGATCACCAAGAAAAACGTGTTC 1860

Query 1861 ATCGGGACCGGCCATCTGCTGGCCAAGATTCTGGTGTCCAGCCTGTACCGGTTCAAGGAC 1920
Sbjct 1861 ATCGGGACCGGCCATCTGCTGGCCAAGATTCTGGTGTCCAGCCTGTACCGGTTCAAGGAC 1920

Query 1921 GTGGCCGAAATCCAGACCAAGGGCTTCCAGACCATCCTGGCTATCCTGAAGCTGTCCGCC 1980
Sbjct 1921 GTGGCCGAAATCCAGACCAAGGGCTTCCAGACCATCCTGGCTATCCTGAAGCTGTCCGCC 1980

Query 1981 AGCTTCTCCAAGCTGCTGGTGCATCACAGCTTCGACCTCGTGATCTTTCACCAGATGAGC 2040
Sbjct 1981 AGCTTCTCCAAGCTGCTGGTGCATCACAGCTTCGACCTCGTGATCTTTCACCAGATGAGC 2040

Query 2041 AGCAACATCATGGAACAGAAGGACCAGCAGTTCCTGAACCTGTGCTGCAAGTGCTTCGCC 2100
Sbjct 2041 AGCAACATCATGGAACAGAAGGACCAGCAGTTCCTGAACCTGTGCTGCAAGTGCTTCGCC 2100

Query 2101 AAGGTGGCCATGGACGACTACCTGAAGAACGTGATGCTGGAACGGGCCTGCGACCAGAAC 2160
Sbjct 2101 AAGGTGGCCATGGACGACTACCTGAAGAACGTGATGCTGGAACGGGCCTGCGACCAGAAC 2160

Query 2161 AACAGCATCATGGTGGAATGCTTGCTGCTGCTGGGCGCCGACGCCAACCAGGCCAAAGAG 2220
Sbjct 2161 AACAGCATCATGGTGGAATGCTTGCTGCTGCTGGGCGCCGACGCCAACCAGGCCAAAGAG 2220

Query 2221 GGAAGCAGCCTGATCTGCCAAGTGTGCGAGAAAGAGTCCAGCCCTAAGCTGGTGGAACTG 2280
Sbjct 2221 GGAAGCAGCCTGATCTGCCAAGTGTGCGAGAAAGAGTCCAGCCCTAAGCTGGTGGAACTG 2280

Query 2281 CTGCTGAACTCCGGCAGCCGCGAACAGGATGTGCGGAAGGCTCTGACCATCAGCATCGGC 2340
Sbjct 2281 CTGCTGAACTCCGGCAGCCGCGAACAGGATGTGCGGAAGGCTCTGACCATCAGCATCGGC 2340

Query 2341 AAGGGCGACAGCCAGATCATCTCTCTGCTGCTGCGGAGACTGGCCCTGGATGTGGCCAAC 2400
Sbjct 2341 AAGGGCGACAGCCAGATCATCTCTCTGCTGCTGCGGAGACTGGCCCTGGATGTGGCCAAC 2400

Query 2401 AACTCTATCTGCCTGGGCGGCTTCTGTATCGGAAAGGTGGAACCCAGCTGGCTGGGCCCC 2460

Disease Models & Mechanisms • Supplementary information

Sbjct 2401 AACTCTATCTGCCTGGGCGGCTTCTGTATCGGAAAGGTGGAACCCAGCTGGCTGGGCCCC 2460

Query 2461 CTGTTCCCTGACAAGACCAGCAACCTGCGGAAGCAGACCAATATCGCCAGCACCCTGGCC 2520

Sbjct 2461 CTGTTCCCTGACAAGACCAGCAACCTGCGGAAGCAGACCAATATCGCCAGCACCCTGGCC 2520

Query 2521 CGGATGGTCATCAGATACCAGATGAAGTCCGCCGTGGAAGAGGGCACCGCCTCTGGCTCC 2580

Sbjct 2521 CGGATGGTCATCAGATACCAGATGAAGTCCGCCGTGGAAGAGGGCACCGCCTCTGGCTCC 2580

Query 2581 GATGGCAACTTCAGCGAGGACGTGCTGAGCAAGTTCGACGAGTGGACCTTCATCCCCGAC 2640

Sbjct 2581 GATGGCAACTTCAGCGAGGACGTGCTGAGCAAGTTCGACGAGTGGACCTTCATCCCCGAC 2640

Query 2641 AGCAGCATGGACTCCGTGTTCGCCCAGAGCGACGACCTGGATAGCGAGGGCTCTGAGGGC 2700

Sbjct 2641 AGCAGCATGGACTCCGTGTTCGCCCAGAGCGACGACCTGGATAGCGAGGGCTCTGAGGGC 2700

Query 2701 AGCTTCCTCGTGAAGAAGAAGTCCAACTCCATCAGCGTGGGCGAGTTCTACCGGGACGCC 2760

Sbjct 2701 AGCTTCCTCGTGAAGAAGAAGTCCAACTCCATCAGCGTGGGCGAGTTCTACCGGGACGCC 2760

Query 2761 GTGCTGCAGAGATGCAGCCCCAATCTGCAGCGGCACAGCAACAGCCTGGGCCCCATCTTC 2820

Sbjct 2761 GTGCTGCAGAGATGCAGCCCCAATCTGCAGCGGCACAGCAACAGCCTGGGCCCCATCTTC 2820

Query 2821 GACCACGAGGATCTGCTGAAGCGGAAGAGAAAGATCCTGTCCAGCGACGACAGCCTGCGG 2880

Sbjct 2821 GACCACGAGGATCTGCTGAAGCGGAAGAGAAAGATCCTGTCCAGCGACGACAGCCTGCGG 2880

Query 2881 TCTAGCAAGCTGCAGAGCCACATGAGACACAGCGACAGCATCAGCTCCCTGGCCAGCGAG 2940

Sbjct 2881 TCTAGCAAGCTGCAGAGCCACATGAGACACAGCGACAGCATCAGCTCCCTGGCCAGCGAG 2940

Query 2941 AGAGAGTACATCACAAGCCTGGATCTGAGCGCCAATGAGCTGCGGGACATCGATGCCCTG 3000

Sbjct 2941 AGAGAGTACATCACAAGCCTGGATCTGAGCGCCAATGAGCTGCGGGACATCGATGCCCTG 3000

Query 3001 AGCCAGAAATGCTGCATCAGCGTGCACCTGGAACATCTGGAAAAACTGGAACTGCACCAG 3060

Sbjct 3001 AGCCAGAAATGCTGCATCAGCGTGCACCTGGAACATCTGGAAAAACTGGAACTGCACCAG 3060

Query 3061 AACGCACTGACCAGCTTCCCTCAGCAGCTGTGCGAGACTCTGAAGTCCCTGACCCATCTG 3120

Sbjct 3061 AACGCACTGACCAGCTTCCCTCAGCAGCTGTGCGAGACTCTGAAGTCCCTGACCCATCTG 3120

Query 3121 GATCTGCATAGCAACAAGTTCACATCCTTCCCCAGCTACCTGCTGAAGATGAGCTGTATC 3180

Sbjct 3121 GATCTGCATAGCAACAAGTTCACATCCTTCCCCAGCTACCTGCTGAAGATGAGCTGTATC 3180

Query 3181 GCCAACCTGGACGTGTCCCGGAACGACATCGGACCCAGCGTGGTGCTGGACCCTACCGTG 3240

Sbjct 3181 GCCAACCTGGACGTGTCCCGGAACGACATCGGACCCAGCGTGGTGCTGGACCCTACCGTG 3240

Query 3241 AAGTGCCCCACCCTGAAGCAGTTCAACCTGAGCTACAACCAGCTGAGCTTCGTGCCCGAG 3300

Sbjct 3241 AAGTGCCCCACCCTGAAGCAGTTCAACCTGAGCTACAACCAGCTGAGCTTCGTGCCCGAG 3300

Query 3301 AACCTGACCGACGTGGTGGAAAAGCTGGAACAGCTGATCCTGGAAGGCAACAAGATCAGC 3360
Sbjct 3301 AACCTGACCGACGTGGTGGAAAAGCTGGAACAGCTGATCCTGGAAGGCAACAAGATCAGC 3360

Query 3361 GGCATCTGTAGCCCCCTGAGACTGAAAGAGCTGAAGATTCTGAATCTGAGCAAGAACCAC 3420
Sbjct 3361 GGCATCTGTAGCCCCCTGAGACTGAAAGAGCTGAAGATTCTGAATCTGAGCAAGAACCAC 3420

Query 3421 ATCTCCAGCCTGAGCGAGAATTTCCTGGAAGCTTGCCCCAAGGTGGAAAGCTTCAGCGCC 3480
Sbjct 3421 ATCTCCAGCCTGAGCGAGAATTTCCTGGAAGCTTGCCCCAAGGTGGAAAGCTTCAGCGCC 3480

Query 3481 CGGATGAACTTCCTGGCCGCCATGCCTTTTCTGCCCCCCAGCATGACCATTCTGAAACTG 3540
Sbjct 3481 CGGATGAACTTCCTGGCCGCCATGCCTTTTCTGCCCCCCAGCATGACCATTCTGAAACTG 3540

Query 3541 TCCCAGAACAAGTTCTCCTGCATCCCCGAGGCCATCCTGAATCTGCCCCACCTGAGATCC 3600
Sbjct 3541 TCCCAGAACAAGTTCTCCTGCATCCCCGAGGCCATCCTGAATCTGCCCCACCTGAGATCC 3600

Query 3601 CTGGATATGTCCTCCAACGACATCCAGTACCTGCCCGGACCCGCCCACTGGAAAAGCCTG 3660
Sbjct 3601 CTGGATATGTCCTCCAACGACATCCAGTACCTGCCCGGACCCGCCCACTGGAAAAGCCTG 3660

Query 3661 AACCTGAGAGAGCTGCTGTTCAGCCACAACCAGATCTCCATTCTGGACCTGTCTGAGAAG 3720
Sbjct 3661 AACCTGAGAGAGCTGCTGTTCAGCCACAACCAGATCTCCATTCTGGACCTGTCTGAGAAG 3720

Query 3721 GCCTACCTGTGGTCCCGGGTGGAAAAACTGCACCTGTCCCACAACAAGCTGAAAGAGATC 3780
Sbjct 3721 GCCTACCTGTGGTCCCGGGTGGAAAAACTGCACCTGTCCCACAACAAGCTGAAAGAGATC 3780

Query 3781 CCCCCCGAGATCGGCTGCCTGGAAAATCTGACCTCTCTGGATGTGTCCTACAACCTGGAA 3840
Sbjct 3781 CCCCCCGAGATCGGCTGCCTGGAAAATCTGACCTCTCTGGATGTGTCCTACAACCTGGAA 3840

Query 3841 CTGCGGAGCTTCCCCAACGAGATGGGCAAGCTGTCTAAGATCTGGGACCTGCCCCTGGAC 3900
Sbjct 3841 CTGCGGAGCTTCCCCAACGAGATGGGCAAGCTGTCTAAGATCTGGGACCTGCCCCTGGAC 3900

Query 3901 GAGCTGCACCTGAACTTCGACTTCAAGCACATCGGCTGCAAGGCCAAGGACATCATCCGG 3960
Sbjct 3901 GAGCTGCACCTGAACTTCGACTTCAAGCACATCGGCTGCAAGGCCAAGGACATCATCCGG 3960

Query 3961 TTTCTGCAGCAGAGGCTGAAGAAAGCCGTGCCCTACAACAGAATGAAGCTGATGATCGTG 4020
Sbjct 3961 TTTCTGCAGCAGAGGCTGAAGAAAGCCGTGCCCTACAACAGAATGAAGCTGATGATCGTG 4020

Query 4021 GGCAATACCGGCTCCGGCAAGACAACCCTGCTGCAGCAGCTGATGAAGACCAAGAAATCC 4080
Sbjct 4021 GGCAATACCGGCTCCGGCAAGACAACCCTGCTGCAGCAGCTGATGAAGACCAAGAAATCC 4080

Query 4081 GACCTGGGCATGCAGTCCGCCACCGTGGGAATCGACGTGAAGGACTGGCCCATCCAGATC 4140

Disease Models & Mechanisms • Supplementary information

Sbjct 4081 GACCTGGGCATGCAGTCCGCCACCGTGGGAATCGACGTGAAGGACTGGCCCATCCAGATC 4140

Query 4141 CGGGACAAGCGGAAGCGGGATCTGGTGCTGAACGTGTGGGACTTCGCCGGCAGAGAAGAG4200
Sbjct 4141 CGGGACAAGCGGAAGCGGGATCTGGTGCTGAACGTGTGGGACTTCGCCGGCAGAGAAGAG4200

Query 4201 TTCTACAGCACCCACCCCCACTTCATGACCCAGCGGGCCCTGTATCTGGCCGTGTACGAC 4260
Sbjct 4201 TTCTACAGCACCCACCCCCACTTCATGACCCAGCGGGCCCTGTATCTGGCCGTGTACGAC 4260

Query 4261 CTGAGCAAGGGCCAGGCCGAAGTGGACGCTATGAAGCCCTGGCTGTTCAACATCAAGGCC 4320
Sbjct 4261 CTGAGCAAGGGCCAGGCCGAAGTGGACGCTATGAAGCCCTGGCTGTTCAACATCAAGGCC 4320

Query 4321 AGAGCCAGCAGCTCCCCCGTGATCCTCGTGGGAACACACCTGGATGTGTCTGACGAGAAG 4380
Sbjct 4321 AGAGCCAGCAGCTCCCCCGTGATCCTCGTGGGAACACACCTGGATGTGTCTGACGAGAAG 4380

Query 4381 CAGCGGAAGGCCTGCATGAGCAAGATTACCAAAGAACTGCTGAACAAGCGGGGCTTCCCT 4440
Sbjct 4381 CAGCGGAAGGCCTGCATGAGCAAGATTACCAAAGAACTGCTGAACAAGCGGGGCTTCCCT 4440

Query 4441 GCCATCCGGGACTACCACTTCGTGAACGCCACCGAAGAGAGCGACGCTCTGGCCAAGCTG 4500
Sbjct 4441 GCCATCCGGGACTACCACTTCGTGAACGCCACCGAAGAGAGCGACGCTCTGGCCAAGCTG 4500

Query 4501 AGAAAGACCATCATCAACGAGAGCCTGAATTTCAAGATTCGGGACCAGCTGGTCGTGGGC 4560
Sbjct 4501 AGAAAGACCATCATCAACGAGAGCCTGAATTTCAAGATTCGGGACCAGCTGGTCGTGGGC 4560

Query 4561 CAGCTGATTCCCGACTGCTACGTGGAACTGGAAAAAATCATCCTGAGCGAACGCAAGAAC 4620
Sbjct 4561 CAGCTGATTCCCGACTGCTACGTGGAACTGGAAAAAATCATCCTGAGCGAACGCAAGAAC 4620

Query 4621 GTGCCCATCGAGTTCCCTGTGATCGACCGGAAAAGACTGCTGCAGCTCGTGCGCGAGAAT 4680
Sbjct 4621 GTGCCCATCGAGTTCCCTGTGATCGACCGGAAAAGACTGCTGCAGCTCGTGCGCGAGAAT 4680

Query 4681 CAGCTGCAGCTGGACGAGAACGAGCTGCCCCACGCCGTGCACTTTCTGAACGAAAGCGGC 4740
Sbjct 4681 CAGCTGCAGCTGGACGAGAACGAGCTGCCCCACGCCGTGCACTTTCTGAACGAAAGCGGC 4740

Query 4741 GTGCTGCTGCATTTTCAAGACCCAGCCCTGCAGCTGTCTGACCTGTACTTCGTGGAACCT 4800
Sbjct 4741 GTGCTGCTGCATTTTCAAGACCCAGCCCTGCAGCTGTCTGACCTGTACTTCGTGGAACCT 4800

Query 4801 AAGTGGCTGTGCAAAATCATGGCCCAGATTCTGACCGTGAAGGTGGAAGGCTGCCCTAAG 4860
Sbjct 4801 AAGTGGCTGTGCAAAATCATGGCCCAGATTCTGACCGTGAAGGTGGAAGGCTGCCCTAAG 4860

Query 4861 CACCCTAAGGGCATCATCTCCAGACGGGACGTGGAAAAGTTTCTGTCCAAGAAGAGGAAG 4920
Sbjct 4861 CACCCTAAGGGCATCATCTCCAGACGGGACGTGGAAAAGTTTCTGTCCAAGAAGAGGAAG 4920

Query 4921 TTCCCCAAGAACTATATGACCCAGTACTTCAAACTGCTGGAAAAATTCCAGATCGCCCTG 4980

Disease Models & Mechanisms • Supplementary information

Sbjct 4921 TTCCCCAAGAACTATATGACCCAGTACTTCAAACTGCTGGAAAAATTCCAGATCGCCCTG 4980

Query 4981 CCCATCGGCGAGGAATACCTGCTGGTGCCTAGCAGCCTGTCCGACCACAGACCCGTGATC 5040

Sbjct 4981 CCCATCGGCGAGGAATACCTGCTGGTGCCTAGCAGCCTGTCCGACCACAGACCCGTGATC 5040

Query 5041 GAACTGCCCCACTGCGAGAACTCCGAGATCATCATCAGACTGTACGAGATGCCCTACTTC 5100

Sbjct 5041 GAACTGCCCCACTGCGAGAACTCCGAGATCATCATCAGACTGTACGAGATGCCCTACTTC 5100

Query 5101 CCCATGGGCTTTTGGAGCCGGCTGATCAATAGGCTGCTGGAAATCAGCCCCTACATGCTG 5160

Sbjct 5101 CCCATGGGCTTTTGGAGCCGGCTGATCAATAGGCTGCTGGAAATCAGCCCCTACATGCTG 5160

Query 5161 AGCGGCAGAGAGAGGGCCCTGCGGCCCAATAGAATGTACTGGCGGCAGGGCATCTATCTG 5220

Sbjct 5161 AGCGGCAGAGAGAGGGCCCTGCGGCCCAATAGAATGTACTGGCGGCAGGGCATCTATCTG 5220

Query 5221 AACTGGTCCCCAGAGGCCTACTGCCTCGTGGGCAGCGAAGTGCTGGATAACCACCCCGAG 5280

Sbjct 5221 AACTGGTCCCCAGAGGCCTACTGCCTCGTGGGCAGCGAAGTGCTGGATAACCACCCCGAG 5280

Query 5281 AGCTTTCTGAAGATCACCGTGCCCAGCTGCCGGAAGGGCTGTATCCTGCTGGGACAGGTG 5340

Sbjct 5281 AGCTTTCTGAAGATCACCGTGCCCAGCTGCCGGAAGGGCTGTATCCTGCTGGGACAGGTG 5340

Query 5341 GTGGACCACATCGACTCCCTGATGGAAGAGTGGTTCCCCGGCCTGCTGGAAATTGACATC 5400

Sbjct 5341 GTGGACCACATCGACTCCCTGATGGAAGAGTGGTTCCCCGGCCTGCTGGAAATTGACATC 5400

Query 5401 TGCGGCGAGGGCGAGACACTGCTGAAGAAGTGGGCCCTGTACAGCTTCAACGACGGCGAG5460

Sbjct 5401 TGCGGCGAGGGCGAGACACTGCTGAAGAAGTGGGCCCTGTACAGCTTCAACGACGGCGAG5460

Query 5461 GAACACCAGAAAATTCTGCTGGACGACCTGATGAAGAAGGCCGAAGAGGGCGACCTGCT 5520

Sbjct 5461 GAACACCAGAAAATTCTGCTGGACGACCTGATGAAGAAGGCCGAAGAGGGCGACCTGCTC 5520

Query 5521 GTGAACCCCGATCAGCCCAGACTGACCATCCCCATCTCCCAGATTGCCCCCGACCTGATC 5580

Sbjct 5521 GTGAACCCCGATCAGCCCAGACTGACCATCCCCATCTCCCAGATTGCCCCCGACCTGATC 5580

Query 5581 CTGGCCGACCTGCCCAGAAACATCATGCTGAACAATGACGAGCTGGAATTCGAGCAGGCC 5640

Sbjct 5581 CTGGCCGACCTGCCCAGAAACATCATGCTGAACAATGACGAGCTGGAATTCGAGCAGGCC 5640

Query 5641 CCCGAGTTTCTGCTGGGGGATGGCAGCTTTGGCAGCGTGTACAGAGCCGCCTATGAGGGG 5700

Sbjct 5641 CCCGAGTTTCTGCTGGGGGATGGCAGCTTTGGCAGCGTGTACAGAGCCGCCTATGAGGGG 5700

Query 5701 GAAGAGGTGGCAGTGAAGATCTTTAACAAGCACACCTCCCTGCGGCTGCTGAGACAGGAA 5760

Sbjct 5701 GAAGAGGTGGCAGTGAAGATCTTTAACAAGCACACCTCCCTGCGGCTGCTGAGACAGGAA 5760

Query 5761 CTGGTGGTGCTGTGCCATCTGCATCACCCTAGCCTGATCAGCCTGCTGGCTGCCGGCATC 5820

Disease Models & Mechanisms • Supplementary information

Sbjct 5761 CTGGTGGTGCTGTGCCATCTGCATCACCCTAGCCTGATCAGCCTGCTGGCTGCCGGCATC 5820

Query 5821 AGACCCCGGATGCTCGTGATGGAACTGGCCAGCAAGGGCTCCCTGGACAGACTGCTGCAG 5880

Sbjct 5821 AGACCCCGGATGCTCGTGATGGAACTGGCCAGCAAGGGCTCCCTGGACAGACTGCTGCAG 5880

Query 5881 CAGGACAAGGCCAGCCTGACCAGAACCCTGCAGCACCGGATTGCTCTGCACGTGGCAGAC 5940

Sbjct 5881 CAGGACAAGGCCAGCCTGACCAGAACCCTGCAGCACCGGATTGCTCTGCACGTGGCAGAC 5940

Query 5941 GGCCTGAGATACCTGCACTCCGCCATGATCATCTACAGGGACCTGAAGCCCCACAATGTG 6000

Sbjct 5941 GGCCTGAGATACCTGCACTCCGCCATGATCATCTACAGGGACCTGAAGCCCCACAATGTG 6000

Query 6001 CTGCTGTTTACCCTGTACCCCAACGCCGCCATCATTGCCAAGATCGCCGACTACGGAATC 6060

Sbjct 6001 CTGCTGTTTACCCTGTACCCCAACGCCGCCATCATTGCCAAGATCGCCGACTACTCAATC 6060

Query 6061 GCCCAGTACTGTTGCAGAATGGGCATCAAGACCTCCGAGGGCACCCCCGGCTTTAGAGCC 6120

Sbjct 6061 GCCCAGTACTGTTGCAGAATGGGCATCAAGACCTCCGAGGGCACCCCCGGCTTTAGAGCC 6120

Query 6121 CCTGAAGTGGCCAGAGGCAACGTGATCTATAATCAGCAGGCCGACGTGTACTCCTTCGGA 6180

Sbjct 6121 CCTGAAGTGGCCAGAGGCAACGTGATCTATAATCAGCAGGCCGACGTGTACTCCTTCGGA 6180

Query 6181 CTGCTGCTGTACGACATCCTGACCACCGGCGGCAGAATCGTGGAAGGACTGAAGTTCCCT 6240

Sbjct 6181 CTGCTGCTGTACGACATCCTGACCACCGGCGGCAGAATCGTGGAAGGACTGAAGTTCCCT 6240

Query 6241 AACGAGTTCGATGAGCTGGAAATCCAGGGAAAGCTGCCCGACCCCGTGAAAGAGTATGGC 6300

Sbjct 6241 AACGAGTTCGATGAGCTGGAAATCCAGGGAAAGCTGCCCGACCCCGTGAAAGAGTATGGC 6300

Query 6301 TGCGCCCCTTGGCCCATGGTGGAAAAACTGATCAAGCAGTGCCTGAAAGAAAACCCCCAG 6360

Sbjct 6301 TGCGCCCCTTGGCCCATGGTGGAAAAACTGATCAAGCAGTGCCTGAAAGAAAACCCCCAG 6360

Query 6361 GAACGGCCCACCAGCGCCCAGGTGTTCGATATCCTGAACAGCGCCGAGCTCGTGTGCCTG 6420

Sbjct 6361 GAACGGCCCACCAGCGCCCAGGTGTTCGATATCCTGAACAGCGCCGAGCTCGTGTGCCTG 6420

Query 6421 ACACGGCGGATTCTGCTGCCTAAGAATGTGATCGTGGAATGCATGGTGGCCACACACCAC 6480

Sbjct 6421 ACACGGCGGATTCTGCTGCCTAAGAATGTGATCGTGGAATGCATGGTGGCCACACACCAC 6480

Query 6481 AACAGCCGGAACGCCAGCATCTGGCTGGGATGCGGCCACACCGATAGAGGCCAGCTGTCC 6540

Sbjct 6481 AACAGCCGGAACGCCAGCATCTGGCTGGGATGCGGCCACACCGATAGAGGCCAGCTGTCC 6540

Query 6541 TTCCTGGACCTGAACACCGAGGGCTACACCAGCGAGGAAGTGGCCGACTCCAGAATCCTG 6600

Sbjct 6541 TTCCTGGACCTGAACACCGAGGGCTACACCAGCGAGGAAGTGGCCGACTCCAGAATCCTG 6600

Query 6601 TGTCTGGCACTGGTGCATCTGCCCGTGGAAAAAGAAAGCTGGATCGTGTCCGGCACCCAG 6660

Disease Models & Mechanisms • Supplementary information

Sbjct 6601 TGTCTGGCACTGGTGCATCTGCCCGTGGAAAAAGAAAGCTGGATCGTGTCCGGCACCCAG 6660

Query 6661 AGCGGAACACTGCTCGTGATCAACACCGAGGACGGCAAGAAGCGGCACACCCTGGAAAAG 6720
Sbjct 6661 AGCGGAACACTGCTCGTGATCAACACCGAGGACGGCAAGAAGCGGCACACCCTGGAAAAG 6720

Query 6721 ATGACCGACAGCGTGACCTGCCTGTACTGCAACTCCTTCAGCAAGCAGAGCAAACAGAAA 6780
Sbjct 6721 ATGACCGACAGCGTGACCTGCCTGTACTGCAACTCCTTCAGCAAGCAGAGCAAACAGAAA 6780

Query 6781 AATTTCCTGCTCGTGGGCACCGCCGACGGCAAGCTGGCCATCTTTGAGGACAAGACCGTG 6840
Sbjct 6781 AATTTCCTGCTCGTGGGCACCGCCGACGGCAAGCTGGCCATCTTTGAGGACAAGACCGTG 6840

Query 6841 AAACTGAAGGGCGCTGCCCCTCTGAAGATCCTGAACATCGGCAACGTGTCCACCCCCCTG 6900
Sbjct 6841 AAACTGAAGGGCGCTGCCCCTCTGAAGATCCTGAACATCGGCAACGTGTCCACCCCCCTG 6900

Query 6901 ATGTGCCTGTCAGAGAGCACCAACAGCACCGAGAGAAACGTGATGTGGGGCGGCTGCGGC 6960
Sbjct 6901 ATGTGCCTGTCAGAGAGCACCAACAGCACCGAGAGAAACGTGATGTGGGGCGGCTGCGGC 6960

Query 6961 ACCAAGATTTTCAGCTTCAGCAACGACTTCACCATCCAGAAGCTGATTGAGACACGGACC 7020
Sbjct 6961 ACCAAGATTTTCAGCTTCAGCAACGACTTCACCATCCAGAAGCTGATTGAGACACGGACC 7020

Query 7021 TCCCAGCTGTTCTCCTACGCCGCCTTCAGCGACTCCAACATCATCACCGTGGTGGTGGAT 7080
Sbjct 7021 TCCCAGCTGTTCTCCTACGCCGCCTTCAGCGACTCCAACATCATCACCGTGGTGGTGGAT 7080

Query 7081 ACCGCCCTGTATATCGCCAAGCAGAACTCTCCAGTGGTGGAAGTGTGGGACAAGAAAACC 7140
Sbjct 7081 ACCGCCCTGTATATCGCCAAGCAGAACTCTCCAGTGGTGGAAGTGTGGGACAAGAAAACC 7140

Query 7141 GAGAAGCTGTGTGGACTGATCGACTGTGTGCATTTCCTGAGAGAAGTGACAGTGAAAGAG 7200
Sbjct 7141 GAGAAGCTGTGTGGACTGATCGACTGTGTGCATTTCCTGAGAGAAGTGACAGTGAAAGAG 7200

Query 7201 AACAAAGAAAGCAAGCACAAGATGTCCTACTCTGGCCGCGTGAAAACACTGTGCCTGCAG 7260
Sbjct 7201 AACAAAGAAAGCAAGCACAAGATGTCCTACTCTGGCCGCGTGAAAACACTGTGCCTGCAG 7260

Query 7261 AAAAACACAGCCCTGTGGATCGGCACCGGCGGAGGACACATTCTGCTGCTGGATCTGTCC 7320
Sbjct 7261 AAAAACACAGCCCTGTGGATCGGCACCGGCGGAGGACACATTCTGCTGCTGGATCTGTCC 7320

Query 7321 ACCCGCAGACTGATCAGAGTGATCTACAACTTCTGCAATTCCGTCCGCGTGATGATGACA 7380
Sbjct 7321 ACCCGCAGACTGATCAGAGTGATCTACAACTTCTGCAATTCCGTCCGCGTGATGATGACA 7380

Query 7381 GCACAGCTGGGGAGCCTGAAAAATGTGATGCTGGTGCTGGGCTACAACCGCAAGAATACC 7440
Sbjct 7381 GCACAGCTGGGGAGCCTGAAAAATGTGATGCTGGTGCTGGGCTACAACCGCAAGAATACC 7440

Query 7441 GAGGGAACCCAGAAGCAGAAAGAAATTCAGAGCTGCCTGACTGTGTGGGATATCAACCTG 7500

Disease Models & Mechanisms • Supplementary information

Sbjct	7441	GAGGGAACCCAGAAGCAGAAAGAAATTCAGAGCTGCCTGACTGTGTGGGATATCAACCTG	7500

Query	7501	CCACACGAAGTGCAGAACCTGGAAAAGCACATCGAAGTGCGGAAAGAACTGGCCGAGAAG	7560
Sbjct	7501	CCACACGAAGTGCAGAACCTGGAAAAGCACATCGAAGTGCGGAAAGAACTGGCCGAGAAG	7560

Query	7561	ATGCGGAGAACCAGCGTGGAATGA	7584
Sbjct	7561	ATGCGGAGAACCAGCGTGGAATGA	7584

Disease Models & Mechanisms • Supplementary information
